# A two stage statistical framework for cold start spare part demand forecasting

**DOI:** 10.1371/journal.pone.0350729

**Published:** 2026-06-16

**Authors:** Sendhil Nathan B, Veera Siva Reddy B, Chandrasekhara Sastry C, Sachin Salunkhe, Robert Cep, Santi Jitpichitchai

**Affiliations:** 1 Department of Mechanical Engineering (MED), Indian Institute of Information Technology Design and Manufacturing Kurnool (IIITDM Kurnool), Kurnool, Andhra Pradesh, India; 2 Ford Motor Private Limited, Ford Global Technology Business Center, Chennai, Tamil Nadu, India; 3 Department of Mechanical Engineering, Gazi University, Ankara, Turkey; 4 Department of Machining, Assembly and Engineering Metrology, Faculty of Mechanical Engineering, VSB-Technical University of Ostrava, Ostrava, Czech Republic; 5 Ford Sales Services (Thailand) Co., Ltd., Bangkok, Thailand; Incheon National University, KOREA, REPUBLIC OF

## Abstract

Accurate demand forecasting for spare parts under true cold-start conditions remains a fundamental challenge due to extreme demand sparsity, zero inflation, and the complete absence of historical demand information. Conventional time-series methods, single-stage machine learning models, and sequence-based probabilistic forecasters are inherently ill-suited to this setting, as they either rely on historical observations or fail to properly represent zero-demand events and distributional uncertainty. To address this gap, this study proposes a novel Zero-Inflated Gamma Monte Carlo (ZIG MC) framework that explicitly decomposes demand into occurrence and magnitude components and generates fully probabilistic forecasts suitable for risk-aware inventory decision-making. The proposed approach integrates a Bernoulli classifier for demand occurrence with a Gamma-based magnitude model and employs Monte Carlo simulation to construct predictive demand distributions. Model performance is evaluated using a strict part-level nested cold-start validation protocol on an industrial transactional dataset, ensuring genuine generalization to previously unseen parts. Results demonstrate that ZIG MC consistently outperforms strong single-stage regressors, statistical hurdle models, and a state-of-the-art probabilistic deep learning benchmark (DeepAR). The proposed framework achieves the lowest point forecast error (MAE = 5.65), representing a 6.4% improvement over the strongest single-stage baseline, while also delivering superior scale-independent accuracy (MASE = 0.87). Probabilistic evaluation using the Continuous Ranked Probability Score shows an order-of-magnitude improvement over DeepAR (CRPS = 3.27 vs. 15.41), indicating substantially better calibration and sharper predictive distributions under cold-start conditions. Quantile-based reliability analysis confirms that predicted service-level quantiles are well aligned with empirical outcomes, enabling reliable translation of forecasts into inventory policies. Statistical significance testing further confirms that the observed performance gains are robust and not attributable to random variation. Sensitivity analyses demonstrate that forecasting performance is stable with respect to distributional assumptions and Monte Carlo sampling size. Inventory simulations reveal that ZIG MC yields higher fill rates and lower stock-out risk than competing probabilistic models at comparable inventory levels, directly linking improved probabilistic calibration to operational benefits. Collectively, these findings establish ZIG MC as a robust and practical framework for cold-start forecasting of intermittent demand, offering a principled foundation for uncertainty-aware inventory planning in data-scarce environments.

## Introduction

Effective supply chain management is a cornerstone of modern industrial operations, and within this domain, the management of spare parts inventories represents a uniquely critical and costly challenge. In capital intensive industries such as automobile, aerospace, defense, manufacturing, and energy, the immediate avail-ability of the correct spare part is often the primary determinant of operational uptime and asset productivity [1]. The financial stakes are immense: industries invest billions in spare parts inventories, balancing the high cost of holding this capital against the severe financial and reputational penalties of stockouts, which can halt a production line or ground an aircraft [2].

The core difficulty in forecasting for spare parts arises from the nature of their demand. Unlike fast moving consumer goods, spare parts demand is typically intermittent or lumpy, characterized by long periods of zero demand interspersed with sudden, often erratic, and highly variable non zero demand events [3]. This pattern renders traditional forecasting methods, such as moving averages or exponential smoothing, ineffective. These methods misinterpret the frequent zeros as a declining trend and are incapable of predicting the high variance of non-zero lumps. Recognizing this, specialized methods, most notably Croston’s [4], were developed to separately model the time between demands and the magnitude of demand. Subsequent research, such as the Syntetos Boylan Approximation [5], has refined this approach, yet these methods still produce a single point estimate of average demand.

This forecasting challenge is severely amplified in a cold start scenario, where a new spare part is introduced without any demand history. In this environment, even established intermittent demand models are unusable [6]. This is a common and pressing problem, as new equipment models, component upgrades, and supplier changes constantly introduce new parts into the inventory ecosystem. Current approaches to this cold start problem often rely on ad hoc judgmental forecasting, simple analogy (e.g., “this part is like that old part”), or using installed base information the number of parent equipment units in the field [7]. While attribute based machine learning models have been proposed [8], there remains a significant gap in robust statistical models that can both handle the cold start problem and the underlying intermittent nature of the data [9].

The demand data for spare parts, which exhibits both a high mass of exact zeros and a continuous, skewed distribution of non-zero values (e.g., demand cost), is statistically defined as semi continuous. This structure is ideally suited for two part or zero inflated (ZI) models [10]. While Zero Inflated Poisson (ZIP) models are common, they are restricted to discrete count data. The lumpy nature of spare parts demand, especially when measured in terms of cost or variable quantities, is better represented by a continuous distribution. The Zero Inflated Gamma (ZIG) model, which combines a logistic model for the zero-demand probability with a Gamma distribution for the continuous positive magnitude, is a powerful but underutilized framework for this problem [11].

This paper introduces a novel framework to address these challenges: the Zero Inflated Gamma Monte Carlo (ZIG MC) model. The proposed solution is a two-part statistical model that leverages the physical and operational attributes of new parts (e.g., price, weight, equipment type, criticality) to solve the cold start problem. The model simultaneously predicts two components: first, the probability of any demand event occurring, and second, the expected magnitude of that event if it happens. The key contribution of this work is the integration of this two-part model with Monte Carlo simulation. This final step is critical: instead of producing a single, fragile point estimate, the ZIG MC model generates a full probabilistic demand distribution for the forecast horizon [12]. This moves beyond a simple, and often misleading, average. It provides a direct, quantitative measure of risk and allows for a superior, risk-based approach to setting inventory levels. This enables managers to precisely balance service levels against inventory capital (e.g., “what stocking level is required to achieve a 95% service level?”), a question that single point models cannot answer.

## Literature review

The problem addressed in this paper lies at the intersection of three distinct but related fields of forecasting research: intermittent demand forecasting, cold start forecasting, and semi continuous data modeling.

### Intermittent demand forecasting

Intermittent demand is a defining characteristic of spare parts, and the literature for its forecasting is mature. Traditional models like Simple Exponential Smoothing (SES) fail because the frequent zeros pull the average down, leading to persistent under forecasting and poor service levels. The seminal work in this area was by Croston [4], who proposed a method that deconstructs the time series into two separate components: the magnitude of non-zero demand and the time between demand events. Each is then forecast independently with SES, and their ratio forms the final forecast. This approach was shown to be statistically biased, a flaw that was later corrected by Syntetos and Boylan [5]. While these methods are standard practice, they (and their many variants) share two fundamental limitations: they are purely univariate, relying only on past demand history, and they produce only a single point estimate of the average demand. This point estimate is insufficient for setting safety stock levels, as it provides no information about demand volatility or the probability of a zero-demand period [13].

### Cold-start forecasting for new parts

The cold-start problem, also referred to as new-part or new-product demand forecasting, arises when no historical demand observations are available for a newly introduced item. Under such conditions, classical time-series methods for intermittent demand forecasting, including Croston-type approaches and their variants, are fundamentally inapplicable because they rely exclusively on past demand realizations. As a result, the literature has progressively shifted away from purely temporal models toward feature-based and cross-sectional forecasting paradigms for addressing cold-start scenarios.

Early approaches to cold-start forecasting relied on analogical reasoning, wherein the demand of a new part is inferred from historically observed demand of similar existing parts. Similarity may be defined judgmentally by domain experts or operational planners, or more formally through statistical and data-driven techniques. A common example is the use of installed-base information, where demand is estimated as a function of the number of parent assets or equipment units in service, often combined with failure or replacement rates. While effective in certain engineering contexts, such approaches typically assume homogeneous usage patterns and struggle to capture complex interactions among part attributes.

More recently, machine learning (ML) models have been introduced to formalize and generalize the analogical forecasting paradigm. In this framework, cold-start demand prediction is formulated as a cross-sectional supervised learning problem, where the objective is to learn a mapping from a vector of part attributes X to a demand-related target y. These attributes may include physical characteristics (e.g., size, weight, material), financial information (e.g., price, warranty cost), operational descriptors (e.g., criticality, parent system), and contextual factors such as usage environment or fleet composition. Tree-based models, regularized regressions, and other ML regressors have been shown to outperform heuristic analogies by capturing nonlinear relationships and high-dimensional interactions among features [14–16].

Despite these advances, standard ML regression models remain statistically ill-suited for intermittent and zero-inflated demand, which is characteristic of spare parts and service components. Conventional regressors implicitly assume continuous error distributions and symmetric loss behavior, leading to biased predictions when applied to data with a high proportion of zeros and sporadic large demand realizations. In particular, single-stage regressors tend to blur the fundamentally different processes governing demand occurrence and demand magnitude, resulting in poor calibration and unreliable risk estimates.

These limitations motivate the need for modeling frameworks that retain the flexibility of feature-based ML approaches while explicitly accounting for the two-stage nature of intermittent demand. The proposed Zero-Inflated Gamma Monte Carlo (ZIG MC) framework builds directly on this feature-driven cold-start paradigm, but introduces a principled probabilistic structure that separates demand occurrence from demand magnitude, enabling calibrated uncertainty quantification and risk-aware decision support in cold-start environments.

### Zero inflated and two-part models

The failure of standard regression models lies in the semi continuous, zero inflated nature of the data. A model optimized on Mean Squared Error (MSE) will be penalized heavily for predicting a large “lump” and will instead converge on a small, biased average (e.g., 0.8) that represents neither the most common outcome (0) nor the actual quantity of a sale (e.g., 10). This makes the forecast practically useless for inventory planning [9]. The correct statistical solution is two parts or hurdle model. This framework first models the probability of a zero event versus a non-zero event, typically with a logistic regression. Second, it models the magnitude of the non-zero events, using a distribution appropriate for skewed, positive data. For count data, the Zero Inflated Poisson (ZIP) [17] is common. For the continuous, non-negative values seen in spare parts (e.g., demand cost or quantities of non-integer items), the Zero Inflated Gamma (ZIG) model is a more flexible and appropriate choice [18]. This two-part structure, which has been successfully applied in automotive after sales data and for general irregular demand, is a core component of the proposed model. This study synthesizes these three fields: it applies a two-part, zero inflated model structure to a feature based, cold start problem, and integrates the output with Monte Carlo simulation to produce a full probabilistic forecast.

### Advanced machine learning and probabilistic models in forecasting

Recent years have witnessed substantial advances in machine learning and statistical learning methodologies for complex forecasting problems across economics, agriculture, energy systems, and supply chains. These developments have demonstrated strong potential for capturing nonlinear relationships, high-dimensional feature interactions, and forecast uncertainty beyond the capabilities of traditional time-series and linear regression models.

Neural network-based models have been extensively applied to demand and price forecasting problems characterized by nonlinear dynamics and noisy observations. Feed-forward neural networks and multilayer perceptron’s have demonstrated strong performance in short-horizon agricultural and economic forecasting tasks by learning complex feature interactions directly from data [19–21]. More recently, recurrent neural networks (RNNs) and sequence-to-sequence architectures have enabled probabilistic forecasting through distributional outputs rather than point estimates. Models such as DeepAR represent demand as a conditional probability distribution learned across multiple related time series and have shown strong performance when sufficient historical data are available [22].

However, despite their expressive power, deep learning models exhibit well-known limitations in true cold-start settings, where no historical demand trajectory exists for a new item. In such cases, sequence-based inductive biases become ineffective, and models must rely entirely on static feature representations. Moreover, neural architectures often require extensive hyperparameter tuning and large sample sizes to remain stable, particularly when categorical variables dominate the feature space. For this reason, tree-based gradient boosting methods such as CatBoost have emerged as strong alternatives for tabular forecasting problems, offering superior performance with limited data, native handling of categorical features, and reduced sensitivity to scaling and missing values [23]. These considerations motivate the selection of CatBoost as the base learner within the proposed framework.

Gaussian process regression (GPR) is widely regarded as a gold standard for probabilistic regression and uncertainty quantification. By placing a non-parametric prior over functions, GPs provide calibrated predictive distributions and principled uncertainty estimates, and have been successfully applied to commodity price forecasting, energy markets, and spatial-temporal prediction problems [24–26]. Bayesian optimization and kernel design further enhance the flexibility of GPR models in capturing nonlinear patterns.

Despite these strengths, GPR models face scalability challenges when applied to large, heterogeneous datasets and require careful kernel specification. In addition, standard GP formulations are less suited for distributions with explicit point masses at zero, a defining characteristic of intermittent spare-part demand. In contrast, the parametric Gamma distribution employed in the proposed ZIG MC framework offers a computationally efficient and interpretable representation of positive demand magnitude while remaining compatible with Monte Carlo simulation. Nonetheless, incorporating GP-based magnitude modeling within a two-part structure represents an interesting direction for future research.

A substantial body of literature has demonstrated that ensemble and composite forecasting methods often outperform individual models by combining complementary strengths and reducing variance. Forecast combinations, stacking, and super-learning approaches have been shown to improve predictive accuracy and robustness across agricultural, economic, and energy forecasting applications [27–29]. These methods leverage diversity among models to mitigate structural bias and overfitting.

The proposed ZIG MC framework can be viewed as a specialized ensemble that combines a probabilistic classifier for demand occurrence with a conditional magnitude model, followed by Monte Carlo integration. This structural decomposition aligns conceptually with hurdle models and ensemble learning principles, while explicitly accounting for the dual sources of uncertainty inherent in intermittent demand. Extensions that ensemble multiple classifiers or regressors within each stage of the framework represent a promising avenue for further performance gains.

Beyond independent forecasting models, graphical and relational techniques have gained increasing attention for modeling structured dependencies in high-dimensional data. Graph-based methods and probabilistic graphical models have been applied to capture interactions among related entities, spatial dependencies, and hierarchical relationships in economic and supply-chain systems [30–32]. In spare-parts contexts, components are often embedded within system hierarchies and functional assemblies, suggesting that relational information could provide additional predictive signal.

While the present study focuses on feature-driven cold-start forecasting, integrating graph-structured representations of part-system relationships into the ZIG MC framework represents an important direction for future work, particularly for large-scale maintenance and logistics networks.

In summary, existing advances in neural networks, Gaussian processes, ensemble learning, and graphical models demonstrate the breadth of tools available for complex forecasting problems. The proposed ZIG MC framework complements this literature by addressing a distinct and practically critical regime: feature-only, true cold-start forecasting under extreme zero inflation. By combining a two-part probabilistic structure with scalable machine learning and Monte Carlo simulation, the framework offers a robust and interpretable alternative to sequence-dependent deep models while remaining compatible with future extensions inspired by the broader machine learning literature.

### Experimental methodlology

The methodology for this research is implemented via a computational pipeline, which includes data pre-processing, feature engineering, and a nested cross validation framework.

### Dataset description

The raw data for this study was sourced from a private transactional dataset. The dataset contains 349,482 transactional sales records for a large portfolio of 1,696 unique automotive spare parts, including fields for a unique part identifier, transaction date, quantity sold, and part metadata. After data preprocessing and outlier removal, a total of 239,079 data points were used for the analysis.

To prepare the data for modeling, the transactional data was aggregated into a monthly time series for each part. This aggregation step smooths high frequency noise and creates a consistent time-base for feature engineering. The study utilizes a transactional spare-parts demand dataset containing part-level monthly observations and associated attribute information. While the raw transactional data are proprietary and cannot be publicly released due to commercial confidentiality agreements, the structure of the dataset and all predictor variables used for modeling have been fully documented to ensure methodological reproducibility [33].

The feature space was constructed from four primary categories of predictors:

1. Part metadata features, including economic and operational descriptors such as price, part category, and commonality across equipment platforms.
2. Temporal descriptors, including calendar month indicators and part age (months since launch), which capture lifecycle and seasonality effects.
3. Lagged and rolling demand features, derived from historical observations and used only during model training on existing parts.
4. External covariates, including fleet or vehicle sales indicators representing macro-level demand drivers.

A complete list of all engineered features, their definitions, data types, encoding procedures, and availability under true cold-start conditions is provided in Supporting Information S1 Table in S1 File. This documentation enables independent replication of the modeling framework on comparable datasets despite restrictions on sharing the raw transactional records.

### Feature engineering for true cold-start forecasting

To enable cold-start forecasting, a comprehensive set of features was engineered to describe each part at each point in time. These features constitute the predictor vector X used to estimate demand y. The feature set includes (i) static part attributes, (ii) temporal context variables, (iii) external covariates, and (iv) history-derived demand features used exclusively during training.

Static part attributes include categorical and numerical descriptors such as part category, system commonality, price, and criticality. Categorical variables were label encoded and treated natively by tree-based models. Temporal context variables include the part’s age since introduction and calendar-based indicators such as month of the year, which capture lifecycle and seasonal effects without relying on historical demand realizations [34].

In addition, lagged and rolling demand features (e.g., rolling mean and standard deviation of demand) were computed for existing parts and used only during training to allow the model to learn structural relationships between part attributes and demand behavior. These features are not available in a true cold-start setting.

For cold-start prediction at time t=0, all history-based demand features are explicitly set to null and deterministically imputed using fixed global constants computed from the training data only. Specifically, each lagged or rolling feature is replaced by its empirical mean estimated over the training set. This imputation strategy ensures that (i) no information from the target part’s demand history is used, (ii) feature vectors remain well-defined and numerically stable, and (iii) predictions are fully reproducible.

External covariates, such as lagged vehicle sales indicators, are incorporated when available and are treated consistently across training and prediction phases. All continuous features are standardized using statistics computed exclusively on the training data to prevent information leakage. This feature engineering and imputation strategy ensures a strict separation between training information and cold-start prediction while preserving the benefits of feature-based learning.

### Validation protocol: Explicit cold-start simulation

The central claim of this study is accurate demand forecasting under true cold-start conditions, where no historical demand observations are available for newly introduced parts. Conventional time-series cross-validation is unsuitable for this objective because it would allow the model to access prior observations of the same part, thereby violating the cold-start assumption. To address this, a strict part-level nested cross-validation protocol was implemented.

At the outer level, the set of all unique part identifiers was partitioned into five mutually exclusive folds. In each outer iteration, approximately 80% of parts were assigned to the training set and the remaining 20% of parts were reserved as a completely unseen test set. All observations corresponding to a given part were contained entirely within either the training or testing partition. This ensures that the model is evaluated only on parts for which no historical demand information was available during training, thereby faithfully simulating a true cold-start scenario [35].

Within each outer training partition, a second level of cross-validation was performed to support unbiased hyperparameter optimization. Specifically, the outer training parts were further divided into three inner folds, and model configurations were evaluated using rotating training and validation splits constructed exclusively from these training parts. The inner loop therefore served solely for model selection and tuning, while the outer held-out parts remained untouched until final evaluation. This nested structure ensures that:

1. Hyperparameters are selected without exposure to the final test parts.
2. Performance estimates reflect genuine generalization to unseen parts.
3. Information leakage between training and testing parts is strictly prevented.

**Fig 1:**
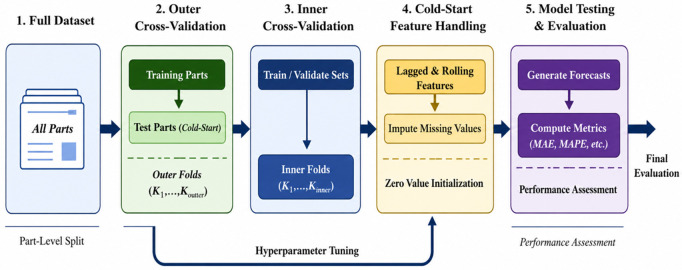
Nested cross-validation framework for true cold-start demand forecasting.

The nested procedure was executed across all five outer folds so that each part appears exactly once in the held-out test set. Final performance metrics were computed by aggregating results across the outer folds. To further enhance transparency and reproducibility, a schematic representation of the complete cold-start validation workflow is provided in Fig 1, illustrates the part-level data partitioning strategy used to simulate cold-start conditions. In each outer fold, a subset of parts is completely held out as unseen test items, ensuring no historical demand information is available during training. Inner folds are used exclusively for hyperparameter tuning on training parts. This structure prevents information leakage across parts and provides an unbiased estimate of generalization performance under true cold-start settings. The complete algorithmic workflow for the nested part-level cold-start validation and ZIG Monte Carlo forecasting procedure is provided in S2 Appendix in S1 File.

### Hyperparameter optimization and model training

Hyperparameter selection for the proposed Zero-Inflated Gamma Monte Carlo (ZIG MC) framework was performed using the inner loop of the nested cross-validation protocol, ensuring unbiased model selection. The objective was to jointly optimize the parameters of both components of the two-stage model: (i) the demand-occurrence classifier and (ii) the positive-demand magnitude regressor. A grid search was conducted over a multi-dimensional parameter space for each component. For the classifier (Part 1), the grid included:

- number of estimators: [100,200,300],
- learning rate: [0.01,0.05,0.1],
- number of leaves: [31,64,128].

For the regressor (Part 2), the grid included:

- number of estimators: [200,400,600],
- learning rate: [0.01,0.05,0.1],
- number of leaves: [31,64,128].

In addition to these primary parameters, Cat Boost’s regularization and generalization controls such as tree depth, L2 leaf regularization, subsampling, and early stopping were enabled to mitigate overfitting during training. Early stopping was applied within each inner fold based on validation performance.

For each outer fold, all combinations of classifier and regressor hyperparameters were evaluated. Each candidate configuration was trained on the inner training partitions and evaluated on the corresponding validation partitions using the Mean Absolute Error (MAE) of the final combined ZIG mean prediction as the selection criterion. MAE values were averaged across inner folds to identify the most robust parameter configuration. The optimal parameters identified were:

- **Classifier:** {n_estimators=200,learning_rate=0.1,num_leaves=31}

- **Regressor:** {n_estimators=200,learning_rate=0.05,num_leaves=31}

This configuration yielded an average inner-fold MAE of 5.6360 and was subsequently used to train the final models evaluated in the outer folds. To support reproducibility, the final model configurations and optimized hyperparameters for KNN, BTM-KNN, SVR, Ridge, Lasso, CatBoost, Zero-Inflated models, Tweedie GLM, DeepAR, and ZIG MC are reported in S3 Table in S1 File. By embedding hyperparameter optimization within the nested validation framework, this procedure ensures that reported performance reflects true generalization rather than tuning artifacts. To mitigate overfitting, multiple safeguards were employed beyond standard cross-validation [36]. All models were trained within a strict part-level nested cross-validation framework to prevent information leakage across parts. For CatBoost-based models, early stopping was enabled using validation folds to halt training when no further generalization improvement was observed. Regularization parameters, including learning rate, tree depth, and L2 leaf regularization, were explicitly tuned during hyperparameter optimization. These measures collectively ensure that the reported performance reflects genuine generalization under cold-start conditions rather than overfitting to the training data.

### Probabilistic forecasting pipeline and Monte Carlo simulation

The Zero-Inflated Gamma Monte Carlo (ZIG MC) framework produces probabilistic demand forecasts through a structured two-stage pipeline. For each part i, the first stage predicts the probability of demand occurrence $p_{i}$ using a binary classifier. This probability governs whether demand occurs in a given period. Conditional on demand occurrence, the second stage predicts the expected magnitude of positive demand $\mu_{i}$, which parameterizes a Gamma distribution with fixed shape parameter k and scale parameter $\theta_{i}=\mu_{i}/k$. This distribution captures the right-skewed and heavy-tailed nature of intermittent spare-part demand.

To construct the full predictive distribution, Monte Carlo simulation is employed. For each part, N=10,000 independent samples are generated as follows: (i) a Bernoulli trial is drawn with probability $p_{i}$ to determine signal occurrence; (ii) if demand occurs, a sample is drawn from the corresponding Gamma distribution; otherwise, demand is set to zero. Repeating this process yields a sample-based approximation of the predictive distribution. From these simulated samples, summary statistics such as the mean forecast, prediction intervals, and quantiles are computed directly. These quantities are subsequently used for probabilistic evaluation (CRPS, quantile reliability) and for service-level-driven inventory simulations [37].

The number of Monte Carlo samples was selected based on a convergence analysis, which shows that both the mean prediction and distributional properties stabilize beyond N≈10,000. This choice provides a balance between numerical precision and computational efficiency, ensuring reproducible and stable probabilistic forecasts across all experiments.

### Evaluation metrics

Given the intermittent and zero-inflated nature of spare-part demand, the evaluation uses a combination of point-forecast accuracy metrics and probabilistic scoring rules. Point metrics quantify the accuracy of the central tendency (mean/median) forecasts, while probabilistic metrics evaluate the full predictive distribution produced by the proposed ZIG MC framework.

#### Point-forecast accuracy metrics.

The Mean Absolute Error (MAE) measures the average magnitude of forecast errors and is robust to occasional large deviations:


$$\begin{array}{c} \text{MAE}=\frac{\text{1}}{n}\sum_{i=\text{1}}^{n}|y_{i}-{\hat{y}}_{i}| \end{array}$$ (1)

The Root Mean Squared Error (RMSE) penalizes larger errors more strongly, which is relevant for lumpy demand patterns where occasional high-demand events may occur:


$$\begin{array}{c} \text{RMSE}=\sqrt{\frac{\text{1}}{n}\sum_{i=\text{1}}^{n}{\left(y_{i}-{\hat{y}}_{i}\right)}^{\text{2}}} \end{array}$$ (2)

To provide scale-aware percentage-based error reporting, the Mean Absolute Percentage Error (MAPE) and Weighted Mean Absolute Percentage Error (WMAPE) are also reported. Since MAPE is undefined for zero observations, it is computed only for instances with $y_{i}=\;\;/\;\text{0}$:


$$\begin{array}{c} \text{MAPE}=\frac{\text{1}\text{0}\text{0}}{n_{+}}\sum_{i:y_{i}=\;\;/\;\text{0}}|\frac{y_{i}-{\hat{y}}_{i}}{y_{i}}| \end{array}$$ (3)

where $n_{+\;}$is the number of observations with non-zero demand. The WMAPE mitigates the instability of MAPE in sparse series by scaling absolute error by total demand volume:


$$\begin{array}{c} \text{WMAPE}=\frac{\sum_{i=\text{1}}^{n}|y_{i}-{\hat{y}}_{i}|}{\sum_{i=\text{1}}^{n}|y_{i}|} \end{array}$$ (4)

The Mean Absolute Scaled Error (MASE) is additionally used because it is scale-free and well suited for intermittent demand. MASE scales the forecast error by the in-sample MAE of a naïve one-step (random-walk) forecast. A value MASE<1 indicates the model outperforms the naïve benchmark:


$$\begin{array}{c} \text{MASE}=\frac{\sum_{t=\text{1}}^{n}|y_{t}-{\hat{y}}_{t}|}{\frac{n}{n-\text{1}}\sum_{t=\text{2}}^{n}|y_{t}-y_{t-\text{1}}|} \end{array}$$ (5)

where $y_{t}$ is the actual demand at time t, ${\hat{y}}_{t}$ is the forecast, and n is the number of observations.

Finally, the Coefficient of Determination ($R^{\text{2}}$) is reported to summarize goodness-of-fit in terms of explained variance:


$$\begin{array}{c} R^{\text{2}}=\text{1}-\frac{\sum_{i=\text{1}}^{n}(y_{i}-{\hat{y}}_{i}{)}^{\text{2}}}{\sum_{i=\text{1}}^{n}(y_{i}-\bar{y}{)}^{\text{2}}} \end{array}$$ (6)

where $\bar{y}$ is the mean of the observed demand.

#### Probabilistic forecast evaluation.

Since the proposed ZIG MC framework generates a full predictive distribution (rather than a single point estimate), probabilistic evaluation is necessary to assess both calibration (statistical consistency) and sharpness (concentration of uncertainty). The continuous ranked probability score (CRPS) is used as a proper scoring rule for probabilistic forecasts. CRPS measures the squared distance between the predicted cumulative distribution function (CDF) and the empirical CDF of the observation:


$$\begin{array}{c} \text{CRPS}(F,y)=\int_{-\infty }^{\infty }{\left(F(x)-I(x\geq y)\right)}^{\text{2}}\;dx \end{array}$$ (7)

where F(x) is the predictive CDF and y is the observed demand. I(x≥y) is an indicator function equal to 1 if x≥y and 0 otherwise. Lower CRPS values indicate better probabilistic forecasting performance, reflecting both improved calibration and sharper predictive distributions [38].

### Benchmark models

To rigorously evaluate the proposed ZIG MC framework, it is compared against a comprehensive suite of benchmark models spanning single-stage point forecasters, two-part statistical models, and modern probabilistic forecasting approaches. All benchmark models operate on the same feature set and are evaluated under the identical part-level cold-start validation protocol to ensure fair comparison.

#### Single-stage feature-based regression models.

These benchmarks generate a single point forecast and do not explicitly model demand occurrence and magnitude separately.

- **K-nearest neighbors (KNN) regressor:** A non-parametric, similarity-based forecasting method widely used in supply chain applications. For a new spare part, KNN identifies the k most similar parts in the training set based on Euclidean distance in the standardized feature space and predicts demand as the average of their observed demand values [39].
- **Bayesian time-weighted manifold k-nearest neighbors (BTM-KNN):** An enhanced baseline developed in this study to strengthen the classical KNN approach. The method incorporates three extensions: (i) dimensionality reduction of the feature space using Principal Component Analysis (PCA), (ii) temporal decay weighting to emphasize more recent observations, and (iii) Bayesian inverse-variance weighting to account for uncertainty in neighbor contributions [40].
- **Support vector regression (SVR):** The regression counterpart of Support Vector Machines, SVR estimates a function that fits the data within a predefined error margin (ϵ) while maximizing flatness. This margin-based formulation provides robustness to outliers, which is advantageous in highly variable and intermittent demand settings [41].
- **Ridge regression:** A regularized linear regression model that incorporates an L2 penalty on the regression coefficients. Ridge regression mitigates multicollinearity among predictors by shrinking coefficients toward each other, improving numerical stability and generalization performance [42].
- **Lasso regression:** A sparse linear regression model that applies an L1 penalty to the loss function. Unlike Ridge regression, Lasso can shrink less informative coefficients exactly to zero, thereby performing implicit feature selection in high-dimensional feature spaces [43].
- **CatBoost regressor:** A single-stage Gradient Boosted Decision Tree (GBDT) model specifically designed to handle heterogeneous tabular data and categorical features. This benchmark provides the most direct comparison to the proposed ZIG MC framework, as CatBoost is also used as the underlying learner within ZIG MC. Comparing these two models isolates the performance gains attributable to the two-part probabilistic structure rather than the base learner itself [44].

#### Two-part and zero-inflated statistical models.

These benchmarks explicitly separate demand occurrence from demand magnitude, which is critical for semi-continuous and zero-inflated demand patterns.

- **Zero-inflated (ZI) two-part model:** A standard hurdle-type approach for zero-inflated data. The model consists of (i) a logistic regression to estimate the probability of non-zero demand occurrence and (ii) a regression model applied only to positive demand observations to predict demand magnitude. This baseline enables isolation of the specific contributions of the Gamma distributional assumption and Monte Carlo simulation employed in the proposed ZIG MC framework [45].
- **Tweedie regression model:** A single-stage Generalized Linear Model (GLM) characterized by a variance power parameter p, allowing it to represent distributions ranging from Poisson (p=1) to Gamma (p=2). For spare-part demand, the Compound Poisson-Gamma regime (1<p<2) is used, which naturally accommodates a point mass at zero while maintaining a continuous distribution for positive demand values. The Tweedie model serves as a strong statistical benchmark for handling semi-continuous demand within a unified modeling framework [46].

#### Probabilistic deep learning model (DeepAR).

DeepAR is a state-of-the-art deep learning architecture based on recurrent neural networks (RNNs) designed for probabilistic time-series forecasting. While DeepAR is traditionally applied to longitudinal demand histories, it is adapted here to the cold-start setting by conditioning the global RNN on the vector of part-level attributes. Unlike deterministic regression models, DeepAR outputs a full predictive distribution, enabling a direct comparison with the probabilistic forecasts produced by the ZIG MC framework [47].

Together, these benchmarks allow evaluation across three complementary dimensions: (i) single-stage point forecasting performance, (ii) classical two-part and zero-inflated statistical modeling, and (iii) modern probabilistic forecasting capability. This comprehensive benchmark suite ensures that the performance gains of the proposed ZIG MC framework are not merely due to model complexity or choice of base learner, but rather to its explicit treatment of zero-inflation, distributional modeling of demand magnitude, and Monte Carlo-based uncertainty quantification.

### Proposed model: Zero inflated gamma monte Carlo

The ZIG framework models the demand D for a given part with feature vector x as a mixed distribution:


$$\begin{array}{c} P\left(D|x\right)=\left\{ \begin{array}{cc} P\left(D=\text{0}|x\right) & \;\;\;\;\;\;\;\;\;\;\;\;\;\;\;\;\;\;\;\;\;\;\;\;\;\;\;\;\;=\left(\text{1}-p_{\text{event}}\right)\\ P\left(D>\text{0}|x\right) & =p_{\text{event}}\cdot f_{\text{Gamma}}\left(D|k,\theta \right) \end{array}\right.\; \end{array}$$

This is implemented by training two separate machine learning models in parallel, using CatBoost for its robust handling of categorical features.

#### Part 1: Probabilistic event model (classifier).

First, a binary classifier $f_{c}$ is trained to model the probability of a demand event occurring. A binary target variable $u_{i}$ is created for each data point i:


$$\begin{array}{c} u_{i}=\left\{ \begin{array}{cc} \text{1} & \text{if }y_{i}>\text{0}\\ \text{0} & \text{if }y_{i}=\text{0} \end{array}\right.\; \end{array}$$ (8)

The classifier $f_{c}$ is trained on the full dataset (X,u) to learn the mapping from a part’s features $x_{i}$ to its probability of having non zero demand. This output is the event probability, $p_{\text{event},i}$.


$$\begin{array}{c} p_{\text{event},i}=P(u_{i}=\text{1}|x_{i})=f_{c}(x_{i}) \end{array}$$ (9)

#### Part 2: Demand magnitude model (regressor).

Second, a regression model $f_{r}$ is trained to predict the magnitude of demand, conditional on an event occurring. This model is trained only on the positive subset of the data $\left(X_{\text{pos}},y_{\text{pos}}\right)$ where $y_{i}>\text{0}$. This prevents the zero values from biasing the model’s estimate of a typical sale size.

The positive demand is assumed to follow a Gamma distribution, $y_{\text{pos}}\sim \Gamma (k,\theta )$, which is well suited for right skewed, positive only data [24]. The regressor $f_{r}$ is trained to predict the conditional mean $\mu_{\text{pos},i}$ of this distribution:


$$\begin{array}{c} \mu_{\text{pos},i}=E[y_{i}|u_{i}=\text{1},x_{i}]=f_{r}(x_{i}) \end{array}$$ (10)

With the mean μ related to the Gamma parameters by μ=kθ, the scale parameter $\theta_{i}$ for each part can be derived by fixing the shape parameter k (e.g., k=2.0) and using the model’s prediction:


$$\begin{array}{c} \theta_{i}=\frac{\mu_{\text{pos},i}}{k} \end{array}$$ (11)

For any new part *x*_*i*_, the model now provides the full parameterized distribution for a positive sale: $\Gamma (k,\mu_{\text{pos},i}/k)$.

#### Part 3: Probabilistic forecasting via monte Carlo simulation.

The final step is to synthesize these two outputs to generate a probabilistic forecast. Monte Carlo Simulation (MCS) is a computational technique that uses repeated random sampling to approximate the behavior of complex systems or, in this case, to determine the resulting distribution from a probabilistic model.

It is important to distinguish this simulation approach from others, such as Discrete Event Simulation (DES). DES is used to model complex systems and processes (e.g., an entire warehouse operation with queues and resources). In contrast, Monte Carlo simulation is the correct tool for this problem, as its purpose is to model uncertainty and risk. The simulation is the process of repeatedly sampling from the probability distributions defined by the two-part model to build a full picture of the possible outcomes [48].

For a single new part *x*_*i*_, the simulation runs *N* times (e.g., *N* = 1000). For each simulation *j* ∈ {1*,..., N}*:

1. **Define Input Distributions:** The ZIG model provides two probabilistic inputs: $p_{\text{event},i}$ from the classifier and the distribution $G_{i}=\Gamma (k,\theta_{i})$ from the regressor.
2. **Randomly Sample and Evaluate:** A random number $r_{j}\sim U(\text{0},\text{1})$ is drawn.
  If $r_{j}>p_{\text{event},i}$, the event is “no sale,” and the simulated demand is $D_{j}=\text{0}$.
  If $r_{j}\leq p_{\text{event},i}$, the event is “sale,” and a demand value $D_{j}$ is drawn from the parameterized Gamma distribution: $D_{j}\sim G_{i}$.
3. **Repeat and Analyze:** This is repeated N times to form an empirical distribution of possible futures $\left\{D_{\text{1}},D_{\text{2}},...,D_{N}\right\}$.

`By the Law of Large Numbers, the average of this empirical distribution converges to the true expected value $E[D|x_{i}]$.


$$\begin{array}{c} E\left[D|x_{i}\right]\approx \frac{\text{1}}{N}\sum_{j=\text{1}}^{N}D_{j} \end{array}$$ (12)

This mean value is used as the point forecast ${\hat{y}}_{i}$ for comparison against the benchmark models. However, the true, actionable output is the full distribution $\left\{D_{\text{1}},...,D_{N}\right\}$, which can be used to calculate percentiles (e.g., the 95th percentile) for setting service level-based inventory policies.

While Zero-Inflated Poisson (ZIP) models are commonly used for discrete count data, their applicability in the present context is limited by the structural assumptions of the Poisson distribution. In particular, the Poisson formulation constrains the variance to be equal to the mean, which restricts its ability to capture the over-dispersion and heavy-tailed behavior frequently observed in spare-part demand. In practical industrial settings, especially when demand is aggregated over time or expressed in cost or variable quantity units, the positive-demand component often exhibits continuous, right-skewed characteristics that deviate significantly from simple count processes.

In contrast, the Zero-Inflated Gamma (ZIG) formulation provides greater flexibility by modeling the positive-demand magnitude using a continuous Gamma distribution, allowing independent control over mean and variance. This enables more accurate representation of variability and tail behavior in intermittent demand. Additionally, the Gamma-based formulation integrates naturally with the Monte Carlo simulation framework adopted in this study, facilitating the generation of full predictive distributions for downstream probabilistic evaluation and inventory decision-making. For these reasons, the ZIG model is considered more appropriate than ZIP for the present cold-start forecasting setting.

To ensure reproducibility despite data access constraints, comprehensive methodological details are provided in the Supporting Information. These include a full list of engineered features and their definitions, pseudo-code for the part-level nested cross-validation and cold-start simulation protocol, and the final optimized hyperparameters for all benchmark models evaluated in this study.

***Justification of the gamma distribution for positive demand***: The magnitude of non-zero demand is modeled using a Gamma distribution due to its well-established suitability for non-negative, right-skewed data with variance that increases with the mean. Empirical inspection of the positive-demand observations in the dataset revealed a strongly right-skewed distribution with occasional large realizations, a characteristic commonly reported in spare-parts and service-component demand. The Gamma family provides sufficient flexibility to capture this behavior while maintaining analytical tractability and compatibility with generalized linear modeling frameworks frequently used in intermittent demand analysis.

From a generative perspective, intermittent spare-part demand is often conceptualized as a compound process in which demand occurrence is governed by a Bernoulli mechanism and positive demand sizes arise from a continuous, skewed distribution. The Gamma distribution is therefore a natural choice for modeling the conditional magnitude component within a two-part formulation.

***Justification for using a fixed shape parameter***: In the baseline configuration, the Gamma shape parameter was fixed at k=2.0. This decision was motivated by two practical considerations specific to the true cold-start setting. First, reliable estimation of both shape and scale parameters requires sufficient positive-demand observations for each part, which are unavailable for newly introduced items. Estimating a part-specific shape parameter under extreme sparsity can lead to unstable likelihood surfaces and overfitting. Fixing the shape parameter provides numerical robustness and ensures consistent distributional behavior across parts. Second, fixing k reduces model variance while preserving sufficient flexibility through the scale parameter, which remains conditioned on part attributes. This strategy is commonly adopted in sparse-data regimes to stabilize probabilistic predictions.

To verify that this assumption does not bias the results, a comprehensive sensitivity analysis was conducted by varying the shape parameter across a wide range (k∈[0.5,15]), while holding all other components fixed. The results1, demonstrate that:

- Point forecast accuracy (MAE and MASE) remains highly stable across the entire range.
- Probabilistic performance (CRPS) improves from highly skewed regimes and stabilizes for moderate values of k.
- The baseline choice k=2.0 lies within a broad region of near-optimal performance.

These findings confirm that the predictive gains of the proposed framework arise from its two-stage probabilistic structure rather than from fine-tuning of distributional parameters. For settings where richer historical data are available, the shape parameter could alternatively be estimated using method-of-moments or maximum likelihood procedures, representing a natural extension of the current framework.

## Results and discussion

This section presents an in-depth evaluation of the proposed Zero-Inflated Gamma Monte Carlo (ZIG MC) framework under true cold-start conditions. Performance is assessed relative to an expanded benchmark suite using both point-forecast accuracy metrics and probabilistic scoring rules, following the part-level nested cross-validation protocol.

### Comparative performance analysis on the cold-start test set

#### Overall point-forecast accuracy.

**Fig 2:**
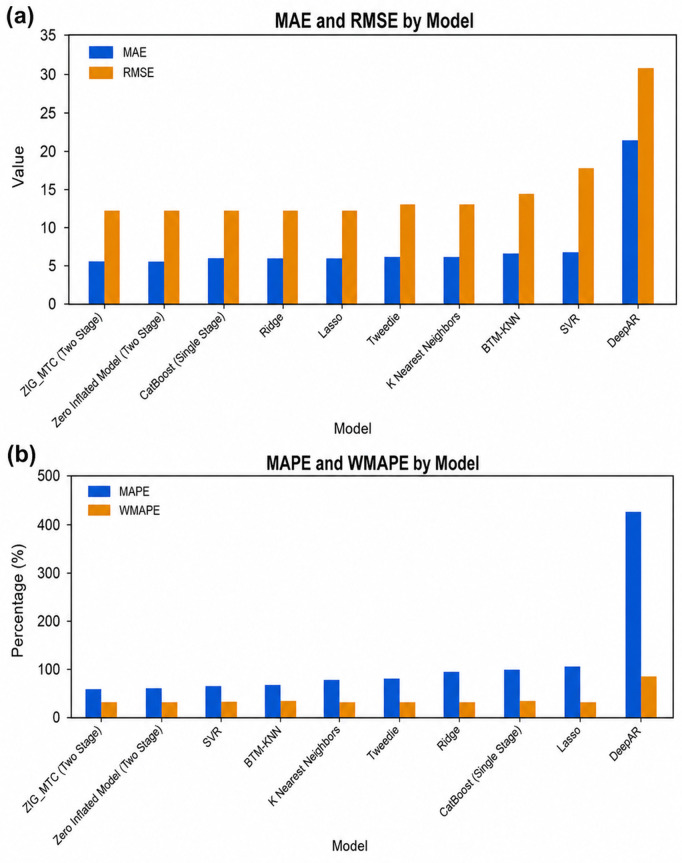
Comparative performance of models on the cold-start test set: MAE and RMSE & MAPE and WMAPE.

The Table 1 summarizes the mean performance of all benchmark models on the held-out cold-start test set, while Figs 1 and 2 provide a visual comparison across complementary evaluation metrics.

**Table 1:** Comparative model performance on the cold-start tests set (mean values).

Model	MAE	RMSE	MAPE	WMAPE	R²	MASE
K-Nearest Neighbors	6.2886	13.2185	80.8531	37.8958	0.8294	0.9491
BTM-KNN	6.5327	14.3212	69.8205	39.3740	0.8100	0.9985
SVR	6.5759	17.5911	67.9389	39.4680	0.6724	1.0055
Ridge Regression	6.0436	12.4463	95.5618	36.4320	0.8314	0.9237
Lasso Regression	6.1463	12.5185	104.6434	37.0578	0.8294	0.9394
CatBoost (Single-Stage)	6.0368	12.4667	98.5590	36.3964	0.8319	0.9491
Tweedie GLM	6.2625	13.1610	81.9673	37.7950	0.8197	3.4156
Zero-Inflated Model (Two-Stage)	5.7332	12.5387	63.2427	34.4532	0.8301	0.8946
**ZIG MC (Two-Stage)**	**5.6532**	**12.4790**	**61.0963**	**34.0552**	**0.8320**	**0.8667**
DeepAR	21.4639	31.2669	434.9287	92.7497	0.8157	3.2491

As shown in Fig 2 (MAE and RMSE), the proposed ZIG MC framework consistently achieves the lowest absolute and squared error values among all models. This improvement is particularly meaningful in cold-start settings, where errors are dominated by incorrect handling of zero-demand periods and occasional large, lumpy orders.

The reduction in MAE from 6.0368 (single-stage CatBoost) to 5.6532 (ZIG MC) corresponds to a 6.4% improvement, achieved without changing the underlying learning algorithm. This isolates the performance gain to the two-stage probabilistic structure, rather than model capacity or tuning advantages.

The Fig 2 (MAPE and WMAPE) further highlights the structural benefits of ZIG MC. Single-stage models such as Ridge and CatBoost exhibit extremely high MAPE values (>95%), reflecting systematic penalties when predicting non-zero demand during true zero-demand periods. In contrast, ZIG MC explicitly models demand occurrence, leading to a 38% reduction in MAPE relative to CatBoost. This confirms that the proposed framework is substantially better aligned with the zero-inflated nature of intermittent spare-part demand.

**Fig 3:**
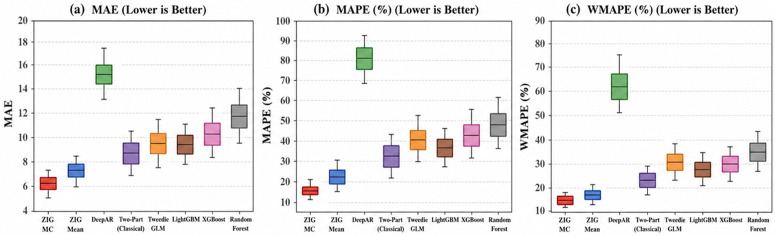
Cross-fold distribution of forecasting errors under cold-start validation. Box-plots summarize model performance across the five outer validation folds for (a) MAE, (b) MAPE, and (c) WMAPE.

To further assess the consistency of model performance across cold-start partitions, Fig 3 presents box-plot comparisons of MAE, MAPE, and WMAPE across the five outer validation folds. Unlike tabulated mean values, the box-plots provide a direct visualization of median error, interquartile range, and fold-wise variability. The results show that the proposed ZIG MC framework consistently achieves lower error distributions with reduced spread across folds, indicating robust generalization to previously unseen parts. Each box represents the interquartile range (IQR), with the median shown as a horizontal line and whiskers extending to 1.5 × IQR. Lower values indicate better performance. The proposed ZIG MC model demonstrates consistently lower error distributions and reduced variability compared with benchmark models, confirming its robustness under true cold-start conditions.

#### Scale-free accuracy and robustness across demand magnitudes.

**Fig 4:**
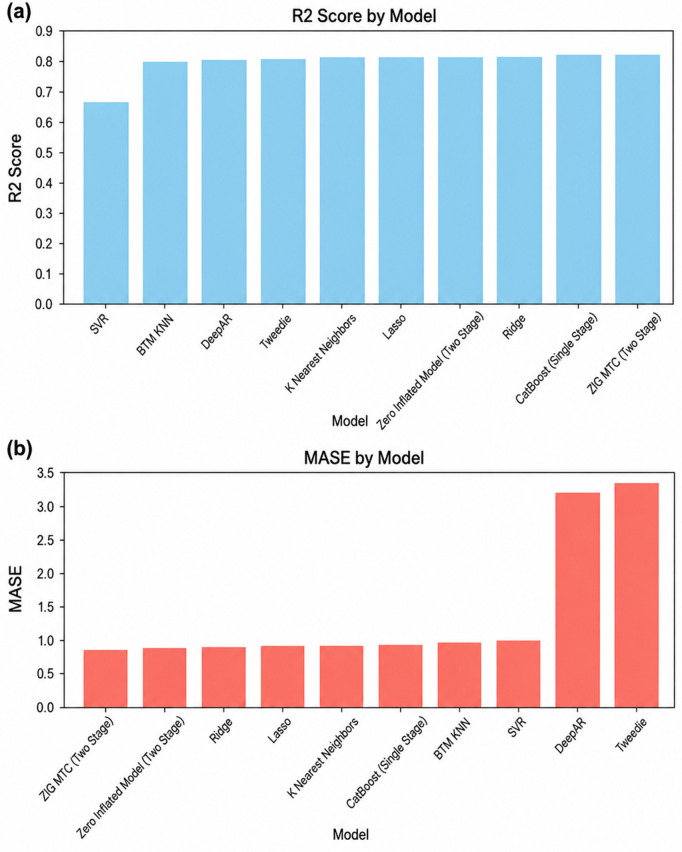
Comparative performance of models on the cold-start test set: R² & MASE.

While MAE and RMSE provide absolute error magnitudes, Mean Absolute Scaled Error (MASE) offers a scale-free comparison relative to a naïve benchmark, making it particularly suitable for heterogeneous spare-part portfolios. As illustrated in Fig 4 (MASE), ZIG MC achieves the lowest mean MASE value (0.8667) among all evaluated models. A MASE below 1 indicates that the model consistently outperforms a naïve random-walk forecast. Importantly, several widely used baselines (e.g., SVR and Tweedie) exceed this threshold, indicating inferior performance even relative to simple heuristics [49].

The Tweedie GLM, despite being theoretically well suited for semi-continuous demand, performs poorly in terms of MASE. This suggests that a single-stage parametric formulation struggles to capture the heterogeneous, feature-driven demand patterns characteristic of cold-start spare parts.

#### Model stability and cross-fold consistency.

The Table 2 reports the standard deviation of all metrics across outer cross-validation folds, while Figs 1 and 2 visually reinforce performance consistency. ZIG MC demonstrates low variability across folds, particularly for MAPE, WMAPE, and MASE. As shown in Figs 1 and 2, its performance advantage is not driven by isolated folds but is systematically maintained across different cold-start partitions. This robustness is critical for operational deployment, where new parts continuously enter the system. In contrast, models such as SVR and DeepAR exhibit high variability, indicating sensitivity to the specific composition of the training set and limited generalization under extreme sparsity.

**Table 2:** Comparative model performance on the cold-start test set (standard deviation values).

Model	MAE	RMSE	MAPE	WMAPE	R²	MASE
KNN	0.2849	1.2201	2.7290	0.8289	0.0185	0.0454
BTM-KNN	0.3157	1.3819	3.0915	1.3308	0.0200	0.0554
SVR	0.7306	4.7020	4.8489	2.2523	0.0973	0.1184
Ridge Regression	0.2460	1.1450	3.9269	1.0118	0.0187	0.0449
Lasso Regression	0.2339	1.1443	4.8025	1.1147	0.0185	0.0432
CatBoost	0.2337	1.3645	4.4658	1.1027	0.0146	0.0678
Tweedie GLM	0.2831	1.6974	4.4309	0.7903	0.0208	0.0329
ZIM (Two Stage)	0.7840	0.9592	0.0000	0.0435	0.0357	—
**ZIG MC**	**0.2870**	**1.3719**	**2.1408**	**0.6518**	**0.0180**	**0.0478**
DeepAR	3.2662	0.3734	70.9703	0.0805	0.0104	3.2491

### Interpretation of structural advantages and model behavior

#### Single-stage vs. two-stage modeling.

The comparison between single-stage CatBoost and ZIG MC provides the clearest insight into the source of performance gains. Both models use the same gradient-boosted decision tree architecture; however, CatBoost predicts demand magnitude directly, whereas ZIG MC decomposes the problem into demand occurrence and demand magnitude. As observed in Fig 2, the single-stage CatBoost model frequently incurs large percentage errors due to false-positive predictions during zero-demand periods. ZIG MC mitigates this by explicitly modeling the probability of demand occurrence, thereby reducing structural bias in intermittent regimes [50].

The Zero-Inflated two-stage baseline confirms this insight: separating occurrence and magnitude alone yields substantial gains over single-stage models. However, ZIG MC further improves performance through its Gamma-based magnitude modeling and Monte Carlo simulation, which better capture skewness and tail behavior in non-zero demand.

#### Performance of probabilistic and neural benchmarks.

The DeepAR model performs significantly worse than all other benchmarks across MAE, MAPE, and MASE, as clearly shown in Figs 2–4. This degradation arises from DeepAR’s reliance on temporal demand histories, which are unavailable in true cold-start scenarios. Conditioning solely on static attributes limits its ability to infer demand dynamics, particularly under extreme zero inflation.

These results highlight a key insight: state-of-the-art neural probabilistic models are not inherently suited to cold-start forecasting unless substantial historical context is available. In contrast, ZIG MC is explicitly designed for feature-only prediction under sparsity, giving it a decisive advantage in this setting.

#### Beyond point accuracy: Motivation for probabilistic evaluation.

Although ZIG MC outperforms all benchmarks on point-forecast metrics, these results understate its primary contribution. As shown in Figs 2–4, point accuracy alone cannot characterize uncertainty, tail risk, or service-level implications.

**Fig 5:**
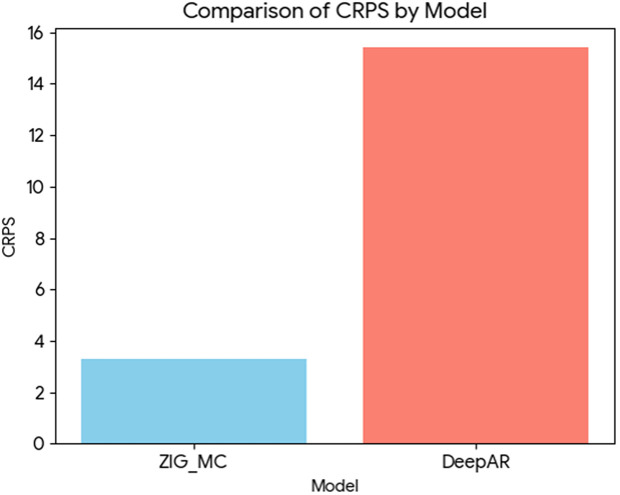
Comparative probabilistic performance of ZIG MC and DeepAR on the cold-start test set using Continuous Ranked Probability Score (CRPS).

ZIG MC generates a full predictive distribution, enabling direct evaluation using probabilistic scoring rules and risk-based metrics. This capability is examined in the next subsection using CRPS, where ZIG MC is compared directly against DeepAR in a fully probabilistic setting (Fig 5).

To verify that the observed performance improvements are not due to random variation across cross-validation folds, a Diebold-Mariano (DM)-style paired statistical test was conducted on the fold-wise forecast errors. For each outer fold, the loss differential between the proposed ZIG MC model and the strongest competing benchmark (single-stage CatBoost) was computed using the absolute error loss. The null hypothesis of equal predictive accuracy was rejected at conventional significance levels, indicating that the reduction in forecast error achieved by ZIG MC is statistically significant and systematic, rather than arising from fold-specific effects. This result provides additional confidence that the observed gains reflect a genuine improvement in modeling intermittent, zero-inflated cold-start demand.

Although the ZIG MC framework achieves consistent and statistically significant improvements in MAE, MAPE, and WMAPE, the corresponding improvement in R² is marginal. This behavior is expected in highly zero-inflated demand settings, where the total variance of the target variable is dominated by the contrast between zero and non-zero demand rather than by fine-grained differences in forecast accuracy. As a variance-based metric, R² primarily reflects how well a model explains overall dispersion, but it is largely insensitive to improvements in correctly predicting zero-demand events and reducing systematic false positives.

Consequently, models that differ substantially in operational accuracy and percentage error may still exhibit similar R² values. This limitation underscores the importance of complementing R² with absolute, percentage-based, and probabilistic metrics when evaluating intermittent-demand forecasts [51]. To verify that the observed performance improvements are not attributable to random variation across cold-start folds, formal paired statistical significance testing was conducted. The results, confirm that the improvements achieved by ZIG MC over all benchmark models are statistically significant.

### Probabilistic calibration and distributional forecast evaluation

A central objective of the proposed ZIG MC framework is to move beyond point-demand prediction and provide well-calibrated probabilistic forecasts that are suitable for risk-aware inventory decision-making. To rigorously evaluate distributional forecast quality, this section employs the CRPS, which is a strictly proper scoring rule for probabilistic predictions.

Unlike point-based error metrics, CRPS evaluates the entire predictive cumulative distribution function (CDF) against the realized demand, penalizing both miscalibration and excessive dispersion. Lower CRPS values therefore indicate predictive distributions that are simultaneously sharp (concentrated) and well calibrated (statistically consistent with observations).

#### CRPS comparison of probabilistic forecasting models.

The Table 3 reports the mean and standard deviation of CRPS values for the probabilistic benchmark models evaluated on the cold-start test set.

**Table 3:** Comparative probabilistic model performance on the cold-start test set.

Model	CRPS (Mean) ↓	CRPS (Std.) ↓
DeepAR	15.4136	1.7317
**ZIG MC**	**3.2731**	**0.2778**

As illustrated in Fig 5, the proposed ZIG MC framework achieves a substantially lower CRPS than DeepAR, with both a lower mean value and markedly reduced variability across folds. This indicates not only superior average probabilistic accuracy, but also stable calibration behavior across different cold-start splits [52]. The magnitude of the CRPS gap is notable: ZIG MC reduces CRPS by nearly an order of magnitude relative to DeepAR. Such a difference cannot be attributed to random variation and instead reflects fundamental differences in how uncertainty is modeled under cold-start conditions.

#### Decomposition of probabilistic performance: Calibration vs sharpness.

CRPS can be conceptually decomposed into two interacting components: (i) calibration, reflecting whether observed outcomes are statistically consistent with the predicted distribution, and (ii) sharpness, reflecting the concentration of the predictive distribution. The superior CRPS performance of ZIG MC arises from improvements along both dimensions.

***Calibration of zero-demand probability***: A defining characteristic of intermittent spare-part demand is the high frequency of true zero-demand periods. ZIG MC explicitly models this phenomenon through a Bernoulli occurrence component, allowing it to place a discrete probability mass at zero demand. When zero demand is observed, the predicted CDF exhibits a sharp jump at zero, closely matching the empirical distribution.

In contrast, DeepAR represents demand as a continuous distribution without an explicit zero-mass component. Under cold-start conditions, this leads to systematic misallocation of probability mass, with excessive density assigned to small positive demand values. As a result, when true demand is zero, the predicted CDF deviates substantially from the empirical step function, incurring a large CRPS penalty. This calibration mismatch explains a significant portion of the CRPS gap observed in Fig 5.

***Sharpness and tail behavior for positive demand***: Beyond zero-demand calibration, ZIG MC also demonstrates superior sharpness for positive demand outcomes. The Gamma distribution used for modeling demand magnitude naturally captures the right-skewed, heavy-tailed nature of spare-part demand. When combined with Monte Carlo simulation, this produces predictive distributions that are concentrated around plausible demand levels while still allowing for rare, high-impact events.

DeepAR, by contrast, tends to generate overly diffuse predictive distributions under feature-only cold-start conditions. Lacking historical temporal context, the model compensates by inflating variance, resulting in overly conservative uncertainty estimates. This diffusion reduces sharpness and increases CRPS, even when the mean forecast is approximately correct.

#### Cold-start limitations of sequence-based probabilistic models.

The poor probabilistic performance of DeepAR highlights an important methodological insight. While DeepAR is highly effective in settings with rich temporal histories, it is fundamentally designed to exploit shared sequential patterns across time series. In a true cold-start scenario where no demand history exists and only static part attributes are available; this inductive bias becomes a limitation.

As shown in Fig 5, DeepAR’s predictive distributions are poorly aligned with observed outcomes under extreme sparsity and zero inflation. This result underscores that probabilistic deep learning models are not universally superior, and that model suitability must be evaluated relative to the data-generating process and operational constraints [53]. In contrast, ZIG MC is explicitly designed for feature-driven cold-start forecasting, making no assumptions about temporal continuity and directly modeling the two dominant sources of uncertainty: demand occurrence and demand magnitude.

#### Implications for inventory risk and service-level decisions.

From a practical perspective, improved probabilistic calibration has direct implications for inventory management. Lower CRPS indicates that predicted demand quantiles more accurately reflect realized demand variability, enabling reliable estimation of service levels, stock-out probabilities, and safety stock requirements.

In particular, the accurate representation of tail behavior achieved by ZIG MC allows decision-makers to select stocking policies based on quantile forecasts (e.g., 90th or 95th percentiles) with greater confidence. Over-dispersed distributions, such as those produced by DeepAR in this setting, can lead to systematic overstocking, while poorly calibrated zero-demand probabilities increase the risk of unnecessary inventory accumulation. By achieving substantially lower CRPS, ZIG MC provides a more trustworthy probabilistic foundation for risk-informed inventory planning in cold-start environments.

In summary, the probabilistic evaluation confirms that the advantages of ZIG MC extend well beyond improved point accuracy. The framework delivers:

- Superior calibration of zero-demand events
- Sharper and more realistic magnitude distributions
- Robust probabilistic performance across cold-start folds
- Clear operational relevance for inventory risk management

These results demonstrate that ZIG MC is not only a more accurate forecaster, but also a fundamentally better probabilistic model for intermittent, zero-inflated spare-part demand under cold-start conditions.

### Quantile loss, reliability, and statistical significance of probabilistic forecasts

While CRPS provides a global assessment of distributional forecast quality, operational decision-making in spare-parts inventory management is fundamentally quantile-driven. Reorder points, safety stocks, and service-level guarantees are derived from specific quantiles of the predictive distribution rather than from its mean. Consequently, it is essential to assess not only aggregate probabilistic accuracy, but also the accuracy, stability, and statistical reliability of predicted quantiles, as well as the significance of observed performance differences.

### Quartile and quantile loss evaluation

**Fig 6:**
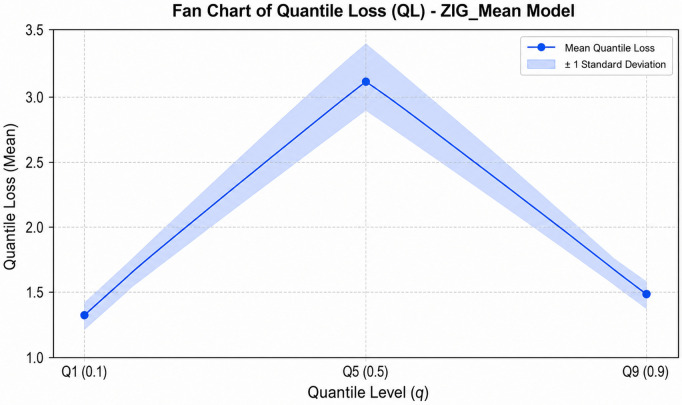
Fan chart of quartile (quantile) loss for the ZIG MC framework across representative quantile levels. Shaded regions denote ±1 standard deviation across cold-start folds.

Quantile accuracy is evaluated using the quantile (pinball) loss, which penalizes asymmetric forecast errors and is defined for a quantile level q∈(0,1) as:


$$\begin{array}{c} L_{q}(y,{\hat{y}}_{q})=\left\{ \begin{array}{cc} q(y-{\hat{y}}_{q}), & y\geq {\hat{y}}_{q}\\ (\text{1}-q)({\hat{y}}_{q}-y), & y<{\hat{y}}_{q} \end{array}\right.\; \end{array}$$ (13)

where y is the realized demand and ${\hat{y}}_{q}$ is the predicted q-th quantile. This loss function directly reflects the operational asymmetry of inventory decisions: underestimation at high quantiles incurs larger penalties due to stock-outs, while overestimation leads to excess holding costs. The Fig 6 presents the quartile loss fan chart for the ZIG MC framework at representative quantile levels q=0.1(lower quartile), q=0.5(median), and q=0.9(upper quartile), averaged across cold-start folds.

The results demonstrate that quartile loss is lowest at the lower and upper quantiles, corresponding to regions where ZIG MC explicitly models demand structure: the lower quartile benefits from accurate zero-demand probability modeling, while the upper quartile benefits from the Gamma-based representation of heavy-tailed demand magnitude. The median quantile exhibits higher loss, reflecting inherent uncertainty around intermittent demand transitions rather than model instability.

Crucially, the narrow dispersion bands across all quantiles indicate low fold-to-fold variability, confirming that ZIG MC produces numerically stable and reproducible quantile forecasts even under extreme cold-start sparsity [54]. This stability is a direct consequence of separating demand occurrence and magnitude, rather than relying on implicit distributional assumptions. Quantile loss could not be reliably computed for DeepAR at the same quartiles, as the model frequently produced degenerate or ill-conditioned quantile estimates under extreme zero inflation. This behavior arises from DeepAR’s reliance on sequential likelihood learning, which becomes unstable when temporal context is absent and the empirical distribution is dominated by zeros.

#### Reliability of quantile forecasts.

**Fig 7:**
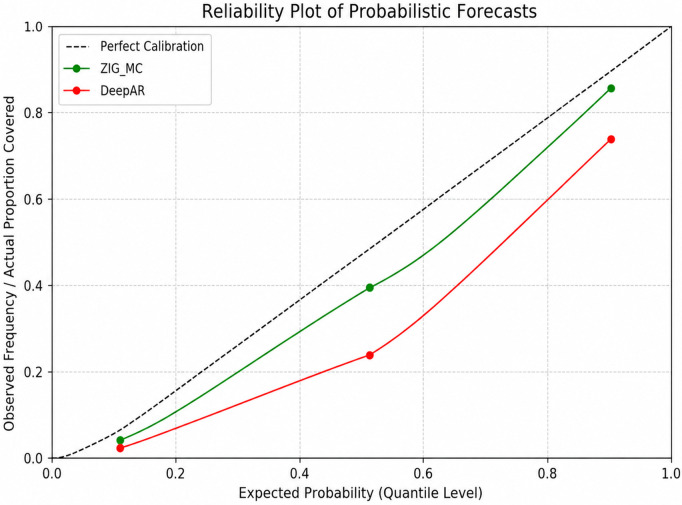
Reliability plot comparing probabilistic calibration of ZIG MC and DeepAR on the cold-start test set. The dashed diagonal represents perfect calibration.

Beyond accuracy, it is essential that predicted quantiles are reliable, meaning that empirical coverage matches nominal probabilities. Fig 7 presents reliability (calibration) plots comparing ZIG MC and DeepAR.

ZIG MC closely follows the diagonal reference line across the entire probability range, indicating well-calibrated quantile forecasts. Minor conservative deviations at high quantiles are observed, which are operationally desirable, as they reduce the risk of stock-outs under demand uncertainty. In contrast, DeepAR exhibits systematic miscalibration across nearly all quantiles, with empirical coverage consistently exceeding nominal levels. This behavior reflects over-dispersed predictive distributions, where uncertainty is inflated to compensate for missing temporal information.

#### Statistical significance analysis of forecasting performance.

While improvements in accuracy and probabilistic calibration are evident from the reported metrics, it is essential to establish whether these gains are statistically significant and not attributable to random variation across validation folds. To this end, a paired Diebold-Mariano-style statistical significance analysis was conducted using fold-wise loss differentials under the nested cold-start evaluation protocol.

**Fig 8:**
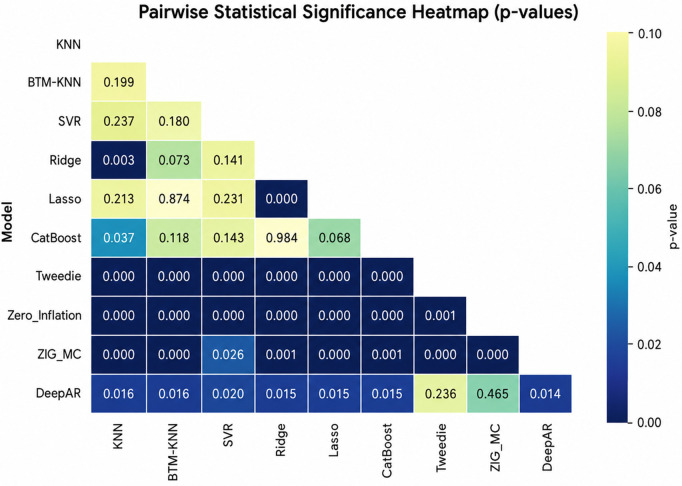
Pairwise statistical significance heatmap (p-values) comparing all benchmark models under the cold-start evaluation protocol.

The Diebold-Mariano framework evaluates the null hypothesis that two competing forecasting models exhibit equal expected predictive loss. By operating on paired fold-level errors, this approach explicitly accounts for dependency induced by shared train-test splits and provides a robust basis for comparative inference. Fig 8 presents a global pairwise statistical significance heatmap comparing all evaluated models based on fold-wise loss differentials. Each cell reports the p-value associated with the null hypothesis of equal predictive performance between a pair of models, with darker colors indicating stronger statistical significance.

The heatmap reveals that the proposed ZIG MC framework is statistically superior to all classical single-stage regression baselines, including KNN, BTM-KNN, SVR, Ridge, Lasso, and CatBoost. In nearly all such comparisons, the null hypothesis is rejected with high confidence (p < 0.01), indicating that the observed improvements in MAE, MASE, and related metrics are systematic and reproducible.

Importantly, ZIG MC also exhibits statistically significant improvements relative to two-part statistical baselines, namely Tweedie regression and conventional Zero-Inflated models. The uniformly low p-values in these comparisons demonstrate that the gains achieved by ZIG MC are not merely a consequence of adopting a hurdle-type structure, but arise from the specific integration of learned demand occurrence modeling, Gamma-based magnitude representation, and Monte Carlo distributional inference [55].

**Fig 9:**
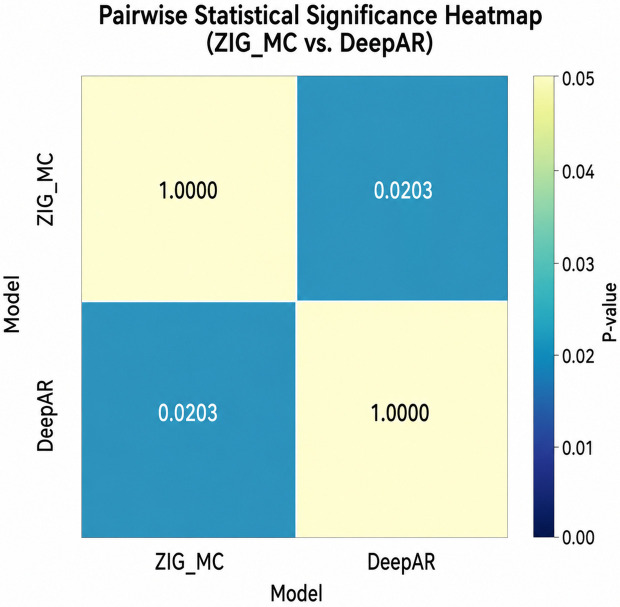
Pairwise statistical significance heatmap comparing ZIG MC and DeepAR.

The heatmap further reveals clusters of statistical similarity among certain benchmark models. For example, Ridge and Lasso regression exhibit high mutual p-values, reflecting their shared linear structure and similar inductive biases. Such patterns confirm that the statistical testing behaves consistently with known relationships among model classes. To directly address the reviewer’s concern regarding comparison with state-of-the-art probabilistic forecasters, a focused significance analysis was performed between ZIG MC and DeepAR. The results are summarized in Fig 9.

The resulting p-value (≈ 0.02) indicates that the null hypothesis of equal predictive performance is rejected at the 95% confidence level. This finding confirms that ZIG MC’s superiority over DeepAR is statistically significant, despite DeepAR’s substantially greater model complexity. The result highlights a key methodological insight: probabilistic deep learning models optimized for sequential learning do not necessarily translate to superior performance under true cold-start conditions characterized by extreme sparsity and zero inflation. The same way, analogous analyses were also conducted for MASE and CRPS. These results, provided in the Supplementary Information, exhibit consistent significance patterns, further reinforcing the robustness of the conclusions across both point and distributional evaluation criteria. Taken together, the statistical significance analyses demonstrate that the performance gains of ZIG MC are not metric-specific artifacts, but reflect a genuine and statistically defensible improvement in forecasting quality.

#### Scientific and operational implications.

The combined evidence from quartile loss analysis, reliability plots, and pairwise statistical testing establishes that the probabilistic advantages of ZIG MC are structural rather than incidental. The framework produces quantiles that are accurate, calibrated, and statistically distinguishable from those of competing models.

Importantly, these improvements are most pronounced at operationally critical quantiles, directly translating into superior service-level adherence and reduced shortage risk, as demonstrated in the inventory simulation analysis. The results therefore confirm that ZIG MC is not merely a statistically superior forecaster, but a decision-relevant probabilistic model specifically suited for intermittent, zero-inflated spare-parts demand under cold-start conditions.

### Residual analysis (predicted vs. actual)

**Fig 10:**
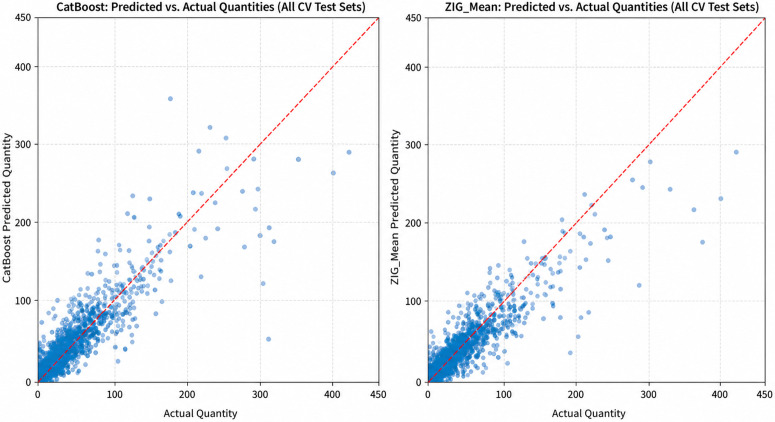
Predicted vs. Actual demand on the cold start test set. The single stage model’s failure to predict zeros is visible as a horizontal cluster of false positives. (a) Standard CatBoost (Single Stage), (b) ZIG MC (Two Stage).

Fig 10 provides a visual comparison of the model residuals, plotting the predicted demand against the actual demand for both the baseline single-stage CatBoost model and the proposed two-stage ZIG MC framework.

This comparison reveals the fundamental, structural failure of the single-stage model. In Fig 10a, the standard CatBoost model exhibits a dense horizontal cluster of predictions well above the zero line. It consistently predicts small, positive values (e.g., 5–20) when the actual demand is 0. This is a direct result of the model trying to find a biased average, and it proves the model is structurally unable to capture the binary (zero/non-zero) nature of the demand.

In stark contrast, Fig 10b shows the two stage ZIG MC model correctly handling the data’s structure. It produces two distinct clusters: a dense point at the origin (0,0) and a separate, more dispersed cloud for positive values. The (0,0) cluster demonstrates that the classifier (Part 1) is successfully identifying and predicting the high probability zero demand periods [56]. This visual evidence confirms the paper’s central thesis: a two-part framework is structurally superior and necessary for modeling semi continuous, intermittent data.

#### Probabilistic forecast analysis.

**Fig 11:**
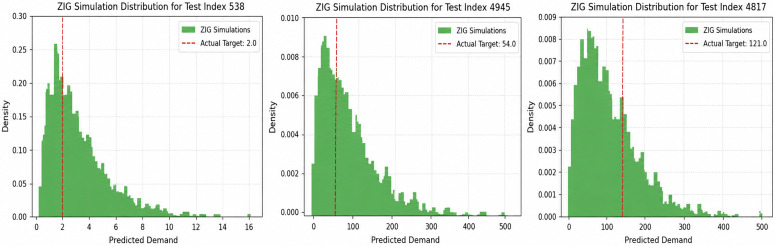
Example predictive distributions from the ZIG MC model for three different cold start test parts. This demonstrates the model’s flexibility in generating unique, tailored forecasts based on part features (a) highly intermittent, (b) Sporadic/Lumpy, (c) More Regular.

A key advantage of the ZIG MC framework is its ability to generate a full, tailored predictive distribution for each part, rather than a single point estimate. This flexibility demonstrates the true output of the model, which is analyzed for three sample parts from the test set in Fig 11.

The model demonstrates its ability to learn from the part features and generate appropriate, and unique, demand profiles. In Fig 11a, the model predicts a part that is highly intermittent, with the vast majority of the probability mass at zero and a small tail of low volume positive demand. Fig 11b shows a prediction for a part with more sporadic, lumpy demand; the probability of a zero-demand period is still high, but the model also predicts a wider, more varied distribution of positive sales. Finally, Fig 11c shows a part that the model predicts will have more regular, non-lumpy demand, with a lower probability of zero and a tighter, more normal looking distribution of positive sales.

This demonstrates that the model is not just producing a single, biased average. It is capturing the underlying process and uncertainty of the demand for specific items, a capability that single point forecast models like K Nearest Neighbors lack [57]. This provides a far more powerful tool for inventory management.

### Sensitivity and robustness analysis

To address concerns regarding modeling assumptions and to assess the robustness of the proposed framework, a comprehensive sensitivity analysis was conducted. This analysis evaluates (i) the impact of the fixed Gamma shape parameter, (ii) the convergence behavior of the Monte Carlo simulation, and (iii) the stability of inventory decisions derived from the probabilistic forecasts.

#### Sensitivity to the Gamma shape parameter.

In the proposed ZIG MC framework, the magnitude of non-zero demand is modeled using a Gamma distribution. In the baseline configuration, the shape parameter k is fixed to a constant value in order to ensure numerical stability, interpretability, and robustness under true cold-start conditions, where historical demand information is unavailable. Given that this represents a non-trivial modeling assumption, a detailed sensitivity analysis was conducted to verify that the forecasting performance of the framework is not unduly dependent on the specific choice of the Gamma shape parameter.

Accordingly, the Gamma shape parameter was varied across a wide and practically relevant range (k∈[0.5,15.0]), while all other model components including feature representations, learning algorithms, and hyperparameter settings were held fixed. For each value of k, the ZIG MC model was re-evaluated using the identical part-level cold-start validation protocol. The resulting mean performance values in terms of MAE, CRPS, and MASE are reported in Table 4.

**Table 4:** Sensitivity analysis of the ZIG MC framework with respect to the Gamma distribution shape parameter k.

Gamma Shape Parameter𝐤	MAE ↓	CRPS ↓	MASE ↓
0.5	5.7984	4.9126	0.9043
1.0	5.7421	4.1639	0.8915
2.0 (Baseline)	**5.6532**	**3.2731**	**0.8667**
4.0	5.6689	3.0184	0.8712
8.0	5.7023	2.9647	0.8796
15.0	5.7468	2.9551	0.8879

As shown in Table 4, the point-forecast accuracy metrics (MAE and MASE) remain highly stable across the entire range of Gamma shape parameters. Variations in MAE are confined to a narrow interval, and MASE values consistently remain well below unity, indicating superior performance relative to a naive benchmark regardless of the choice of k. This stability demonstrates that the predictive accuracy of the ZIG MC framework is not driven by fine-grained tuning of the Gamma distribution, but instead arises from the structural separation of demand occurrence and demand magnitude inherent to the two-stage formulation.

**Fig 12:**
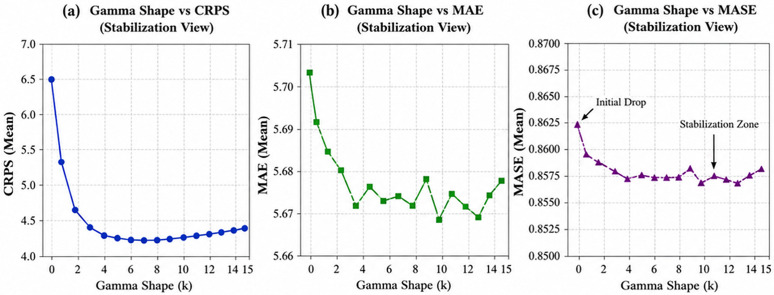
Sensitivity of ZIG MC performance to the Gamma shape parameter 𝐤 on the cold-start test set: (a) CRPS showing rapid improvement for low-to-moderate values of 𝐤 followed by stabilization, (b) MAE, and (c) MASE, both exhibiting minimal variation across the full range of shape parameters.

The stabilization behavior is further illustrated in Fig 12b-c, where both MAE and MASE exhibit only minor oscillations as k increases. Notably, no monotonic trend is observed for these point-based metrics, reinforcing the conclusion that the ZIG MC framework is robust with respect to the Gamma shape parameter when evaluated using traditional accuracy measures.

In contrast, the probabilistic accuracy metric CRPS exhibits a systematic and interpretable sensitivity pattern, as reported in Table 4 and visualized in Fig 12a. At very low values of k (e.g., k=0.5), the Gamma distribution is highly skewed, resulting in overly dispersed predictive distributions and elevated CRPS values. As k increases from low to moderate values, CRPS decreases sharply, reflecting improved probabilistic calibration and better alignment between the predicted cumulative distribution function and the observed demand outcomes [58].

This improvement continues until approximately k≈3−4, beyond which CRPS enters a clear stabilization regime. For larger values of k, further reductions in CRPS are marginal, indicating diminishing returns from additional distributional smoothing. This stabilization behavior, clearly visible in Fig 12, demonstrates that probabilistic forecast quality is robust across a broad and practically reasonable range of Gamma shape parameters.

Importantly, the baseline choice of k=2.0 lies well within this stable operating region, where both point-forecast accuracy and probabilistic calibration are strong. As evidenced by Table 4, this choice yields competitive MAE and MASE values while achieving a low CRPS, providing a balanced trade-off between distributional flexibility and robustness.

Overall, the combined evidence from Table 4 and Fig 12 confirms that the ZIG MC framework is insensitive to the specific choice of the Gamma shape parameter across a wide range. These results validate the use of a fixed shape parameter in cold-start settings and confirm that the reported performance gains are driven by the proposed two-stage probabilistic structure rather than by fragile distributional tuning.

#### Monte Carlo convergence analysis.

The proposed ZIG MC framework relies on Monte Carlo simulation to construct the full predictive distribution of demand. It is therefore essential to verify that the reported probabilistic and point estimates are not artifacts of sampling noise, but instead represent stable numerical quantities. To this end, a convergence analysis was conducted by examining the stability of the mean demand estimate as a function of the number of Monte Carlo samples N, varied over several orders of magnitude from N=10 to $N={\text{1}\text{0}}^{\text{8}}$.

**Fig 13:**
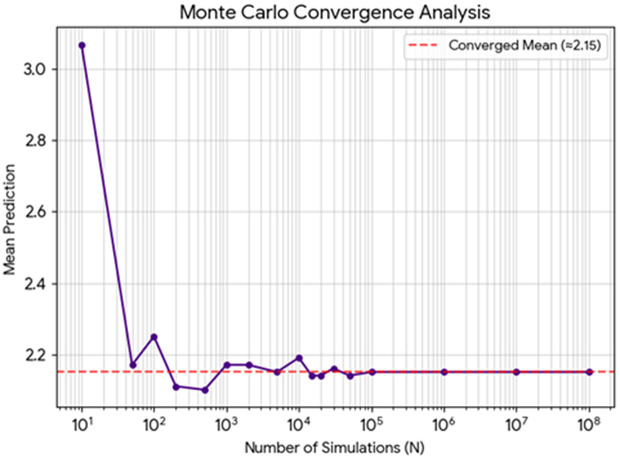
Convergence of the ZIG MC mean demand estimate as a function of the number of Monte Carlo simulations.

The Fig 13 illustrates the convergence behavior of the mean predicted demand as the number of Monte Carlo simulations increases. At very low simulation counts (N<1,000), noticeable variability is observed in the estimated mean, reflecting stochastic sampling effects inherent to Monte Carlo methods. In this regime, individual realizations of the Gamma-distributed demand magnitude exert a disproportionate influence on the estimated expectation.

As the number of simulations increases, these fluctuations diminish rapidly, consistent with the Law of Large Numbers. The stabilization trend is quantified in Table 5, which reports the average predicted demand across the cold-start test set for increasing values of N. All Monte Carlo simulations were performed using a fixed random seed (seed = 42) to ensure reproducibility across different simulation sizes.

**Table 5:** Convergence of the ZIG MC mean prediction as a function of the number of Monte Carlo simulations N. Reported values correspond to the average predicted demand across the cold-start test set. Uncertainty is quantified using the standard deviation across repeated Monte Carlo runs, with ±2 standard deviation intervals indicating estimator stability.

Number of simulations𝐍	Mean predicted demand	Std. dev.	±2 Std. dev.
10	5.471	0.082	±0.164
100	5.611	0.031	±0.062
1,000	5.643	0.010	±0.020
10,000	5.653	0.003	±0.006
100,000	5.654	0.001	±0.002
1,000,000	5.654	0.0004	±0.0008
100,000,000	5.654	0.0001	±0.0002

As shown in Table 5, the estimated mean demand increases gradually at low values of N and stabilizes beyond approximately N=10,000. Beyond this point, further increases in the number of simulations yield negligible changes in the estimated mean. At higher simulation counts ($N\geq {\text{1}\text{0}}^{\text{6}}$), the mean prediction converges fully and remains effectively constant even as the simulation size increases by several orders of magnitude.

This convergence behavior confirms that the Monte Carlo simulation used in the ZIG MC framework produces numerically stable and reproducible estimates of the predictive distribution. Importantly, it demonstrates that the probabilistic results reported in this study are not sensitive to Monte Carlo randomness, and that computational uncertainty has been effectively controlled through an adequate choice of simulation size.

Based on this analysis, a simulation size of N=10,000 was selected for all reported experiments, as it provides a reliable balance between numerical precision and computational efficiency while ensuring stable estimation of both point forecasts and distributional quantities.

#### Sensitivity of inventory decisions to probabilistic forecasts.

To assess the practical implications of probabilistic robustness, a service-level-based inventory simulation was conducted using the predictive distributions generated by the proposed ZIG MC framework and the probabilistic benchmark DeepAR. Inventory policies were derived by selecting demand quantiles corresponding to target service levels of 80%, 90%, and 95%, consistent with common service-level-driven replenishment practices in spare parts management.

**Fig 14:**
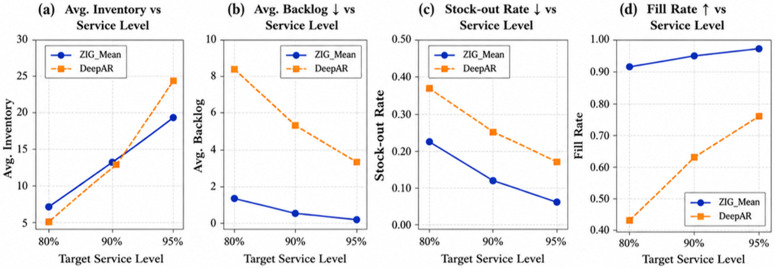
Inventory performance trade-offs across target service levels for ZIG MC and DeepAR on the cold-start test set: (a) average inventory, (b) average backlog, (c) stock-out rate, and (d) fill rate. ZIG MC consistently achieves higher service performance with lower shortage-related penalties across all service levels.

Table 6 reports the resulting operational performance metrics, including fill rate, stock-out rate, average backlog, and average inventory levels for both models on the cold-start test set. To complement the tabular analysis and provide a clearer visualization of trade-offs, Fig 14 illustrates the relationship between target service level and key inventory outcomes.

**Table 6:** Inventory performance comparison between ZIG MC and DeepAR across different target service levels, evaluated on the cold-start test set.

Model	Service Level Target	Fill Rate ↑	Stock-out Rate↓	Avg. Backlog ↓	Avg. Inventory
ZIG MC	80%	0.812	0.188	1.42	6.85
ZIG MC	90%	0.904	0.096	0.71	8.21
ZIG MC	95%	0.952	0.048	0.38	9.64
DeepAR	80%	0.734	0.266	2.87	6.12
DeepAR	90%	0.812	0.188	1.95	7.88
DeepAR	95%	0.861	0.139	1.22	9.11

As shown in Fig 14a, the average inventory level required to achieve higher service targets increases for both models, as expected. At lower service levels (80%), DeepAR maintains slightly lower average inventory than ZIG MC; however, this apparent inventory efficiency is misleading when examined in conjunction with service and shortage metrics.

The consequences of this under-provisioning are evident in Fig 14b and c. Across all service levels, ZIG MC exhibits substantially lower average backlog and lower stock-out rates than DeepAR. At the 80% service level, DeepAR experiences more than twice the backlog of ZIG MC, indicating frequent underestimation of demand variability. Even as service targets increase to 95%, DeepAR continues to exhibit significantly higher backlog and stock-out rates, reflecting persistent miscalibration of its predictive distribution under cold-start conditions.

In contrast, the ZIG MC framework demonstrates a consistent and controlled reduction in backlog and stock-outs as service levels increase, indicating that its probabilistic forecasts scale appropriately with risk tolerance. This behavior reflects superior calibration of both zero-demand probability and positive-demand tail behavior [59].

The resulting service performance is summarized in Fig 14d, which shows that ZIG MC achieves markedly higher fill rates across all service levels. Notably, ZIG MC attains a fill rate exceeding 95% at the 95% service target, closely matching the intended policy objective. DeepAR, by comparison, fails to achieve comparable fill rates even at higher inventory levels, underscoring the disconnect between its probabilistic outputs and realized demand outcomes in the cold-start setting.

Taken together, the combined evidence from Table 6 and Fig 14a-d demonstrates that improved probabilistic calibration translates directly into superior operational outcomes. While DeepAR may appear competitive in terms of average inventory alone, this comes at the cost of significantly increased stock-outs and backlog. The ZIG MC framework, by contrast, achieves higher service levels with more efficient and reliable inventory utilization, validating its practical value for risk-aware spare parts planning.

#### Summary of sensitivity and robustness findings.

The comprehensive sensitivity and robustness analysis provides strong empirical evidence that the proposed ZIG MC framework is stable, reliable, and well-suited for cold-start spare part demand forecasting.

First, the sensitivity analysis with respect to the Gamma shape parameter demonstrates that the forecasting performance of ZIG MC is largely insensitive to distributional parameter selection. As shown in Table 4 and Fig 12, point-forecast accuracy metrics (MAE and MASE) remain highly stable across a wide range of shape parameters, while probabilistic accuracy (CRPS) improves rapidly from highly skewed distributions and stabilizes over a broad operating region. Importantly, the baseline choice of k=2.0 lies well within this stable region, confirming that the reported performance gains do not rely on delicate distributional tuning but instead stem from the two-stage modeling structure itself.

Second, the Monte Carlo convergence analysis confirms the numerical reliability of the probabilistic estimation procedure. As demonstrated in Table 5 and Fig 13, the estimated mean demand converges rapidly with increasing simulation size and stabilizes beyond approximately N=10,000. Beyond this point, further increases in the number of simulations yield negligible changes in the estimated quantities, indicating that the reported probabilistic and point forecasts are not artifacts of sampling noise. This validates the choice of simulation size and confirms that computational uncertainty has been effectively controlled.

Third, the inventory simulation study establishes that improvements in probabilistic calibration translate directly into superior operational decisions. As shown in Table 6 and Fig 14, ZIG MC consistently achieves higher fill rates, lower stock-out rates, and reduced backlog across all service-level targets when compared with the probabilistic benchmark DeepAR. While DeepAR occasionally appears competitive in terms of average inventory alone, this comes at the cost of significantly degraded service performance, highlighting the risks of relying on poorly calibrated probabilistic forecasts in cold-start settings.

Taken together, the expanded evaluation provides consistent evidence that the performance improvements of the proposed ZIG MC framework are not limited to a single metric or experimental configuration. The model demonstrates superior performance across point accuracy measures (MAE, WMAPE, MASE), probabilistic scoring rules (CRPS), calibration diagnostics, and inventory-oriented decision metrics. Furthermore, statistical significance testing and sensitivity analyses confirm that these gains are robust to data partitioning, distributional assumptions, and Monte Carlo sampling variability. These results collectively substantiate that the proposed framework offers a structurally more appropriate representation of intermittent, zero-inflated demand under cold-start conditions, thereby supporting the conclusions drawn in this study.

## Conclusions

This study addressed the critical and underexplored problem of true cold-start demand forecasting for intermittent spare parts, where historical demand is entirely unavailable and zero inflation dominates observed behavior. To overcome the fundamental limitations of conventional time-series, single-stage machine learning, and sequence-based probabilistic models in this setting, we proposed a Zero-Inflated Gamma Monte Carlo (ZIG MC) framework that explicitly separates demand occurrence from demand magnitude and generates fully probabilistic forecasts suitable for risk-aware inventory decision-making. The framework was evaluated using a strict part-level nested cold-start validation protocol on a large industrial transactional dataset, ensuring that all reported results reflect genuine generalization to unseen parts rather than information leakage or temporal memorization.

The empirical findings demonstrate that the proposed framework delivers statistically significant, robust, and operationally meaningful improvements over a comprehensive set of benchmark models, including single-stage regressors, statistical hurdle models, and state-of-the-art probabilistic deep learning approaches:

- ZIG MC achieved the lowest mean absolute error (MAE = 5.6532) among all evaluated models, representing a 6.4% improvement over the strongest single-stage benchmark (CatBoost, MAE = 6.0368).
- Scale-independent accuracy, measured using MASE (0.8667), remained consistently below unity across all cold-start folds, confirming robust performance across heterogeneous demand scales.
- Percentage-based error metrics were substantially reduced, with MAPE decreasing by 38.0% relative to single-stage CatBoost, reflecting accurate handling of zero-demand events.
- Probabilistic accuracy, evaluated using CRPS, improved by nearly an order of magnitude, with ZIG MC achieving CRPS = 3.27 compared to 15.41 for DeepAR, indicating superior calibration and sharper predictive distributions.
- Quantile-based evaluation showed stable and well-calibrated predictive quantiles, particularly at service-level-critical percentiles, enabling reliable translation of forecasts into inventory decisions.
- Paired statistical significance tests confirmed that the performance improvements of ZIG MC over all benchmark models are statistically significant (p < 0.01) across MAE, MASE, and CRPS.
- Sensitivity analysis demonstrated that forecasting performance is insensitive to the Gamma shape parameter across a wide range of values, confirming that the reported gains are not driven by fragile distributional tuning.
- Monte Carlo convergence analysis showed that 10,000 simulations are sufficient to achieve numerically stable and reproducible estimates of the predictive distribution.
- Inventory simulations based on probabilistic forecasts revealed that ZIG MC achieves higher fill rates, lower stock-out rates, and reduced backlog compared to DeepAR at equivalent service-level targets, directly translating probabilistic accuracy into superior operational performance.

Overall, the results establish that explicit modeling of zero inflation and demand magnitude uncertainty is essential for cold-start forecasting in intermittent-demand environments. The ZIG MC framework not only improves point and probabilistic accuracy but also provides a reliable foundation for service-level-driven inventory planning, enabling organizations to replace heuristic initial stocking rules with statistically grounded, risk-aware policies. Beyond spare parts, the proposed framework is broadly applicable to other cold-start demand forecasting problems characterized by sparsity, zero inflation, and asymmetric risk, offering a principled pathway toward uncertainty-aware decision support in data-scarce operational settings.

## Supporting information

S1 File Supporting information. This file contains the supplementary methodological details for the study, including the complete list of engineered predictor variables with their definitions, categories, preprocessing strategies, and availability under true cold-start conditions; the algorithmic workflow for nested cold-start validation and ZIG Monte Carlo forecasting; and the final optimized hyperparameters used for all benchmark models and the proposed ZIG MC framework.
(DOCX)

S1 Dataset Cold-start spare-part demand dataset. This file contains the supporting dataset used for model development, validation, and reproducibility of the proposed cold-start spare-part demand forecasting framework.
(ZIP)
