# Supplementary material for: A two stage statistical framework for cold start spare part demand forecasting
Source: PLoS One. 2026 Jun 16;21(6):e0350729. doi: 10.1371/journal.pone.0350729 (PMC13271490; doi:10.1371/journal.pone.0350729)
Supplement: S1 File — This file contains the supplementary methodological details for the study, including the complete list of engineered predictor variables with their definitions, categories, preprocessing strategies, and availability under true cold-start conditions; the algorithmic workflow for nested cold-start validation and ZIG Monte Carlo forecasting; and the final optimized hyperparameters used for all benchmark models and the proposed ZIG MC framework. (DOCX) [file pone.0350729.s001.docx]

# Supporting information

**S1 Table. Complete list of engineered predictor variables, their categories, definitions, availability under true cold-start conditions, and preprocessing strategy.**

This table provides full transparency regarding the construction of the feature vector $X$used by all benchmark and proposed models.

| **Feature Name** | **Feature Category** | **Description** | **Available at True Cold Start** | **Imputation / Preprocessing Strategy** |
| --- | --- | --- | --- | --- |
| **qty** | Lagged | Monthly aggregated demand quantity for the part | No | Missing values set to 0 |
| **qty_rolling_3m** | Lagged, Temporal | Three-month rolling mean of demand quantity | No | Missing values set to 0 |
| **qty_rolling_6m** | Lagged, Temporal | Six-month rolling mean of demand quantity | No | Missing values set to 0 |
| **qty_rolling_std_3m** | Lagged, Temporal | Three-month rolling standard deviation of demand | No | Missing values set to 0 |
| **months_since_launch** | Temporal, Metadata | Number of months elapsed since part introduction | Yes | Clipped to non-negative values |
| **month_of_year** | Temporal | Calendar month index (1–12) capturing seasonality | Yes | Used as categorical temporal indicator |
| **veh_sales_lag3** | External, Lagged | Vehicle sales volume lagged by 3 months | Yes | Missing values set to 0 |
| **veh_sales_lag6** | External, Lagged | Vehicle sales volume lagged by 6 months | Yes | Missing values set to 0 |
| **price** | Metadata | Median selling price of the part | Yes | Missing values set to 0 |
| **category_enc** | Metadata, Categorical | Encoded part category (label encoding) | Yes | Missing category set to “NA”, then encoded |
| **commonality_enc** | Metadata, Categorical | Encoded commonality indicator across platforms | Yes | Missing category set to “NA”, then encoded |

Lagged and rolling features were used exclusively during model training on existing parts to capture historical demand behavior. For true cold-start prediction (i.e., parts with no prior demand history), these history-dependent features are unavailable and were systematically imputed using zero-value initialization. This design ensures that the deployed model operates strictly on metadata, temporal descriptors, and external covariates, thereby preserving the integrity of the cold-start assumption while preventing information leakage from historical demand signals.

**S2 Appendix. Algorithm for Nested Cold-Start Validation and ZIG Monte Carlo Forecasting**

**Algorithm S1. Nested cross-validation for true cold-start spare-part demand forecasting**

**Inputs:**
Dataset $D$containing part identifiers, monthly demand values, metadata, temporal variables, and external covariates;
set of unique part identifiers $P$;
number of outer folds $K_{\text{outer}}=5$;
number of inner folds $K_{\text{inner}}=3$;
Monte Carlo simulations $N_{\text{MC}}=100000$;
Gamma shape parameter $k=2.0$;
random seed $s=42$;
candidate model configurations $\Theta$.

**Output:**
Aggregated point-forecast metrics, probabilistic metrics, inventory metrics, and predictive samples across all outer folds.

**Step 1. Initialize**

Set random seed $s=42$.

Create an empty results object to store predictions, observed values, predictive samples, and performance metrics from each outer fold.

**Step 2. Outer part-level split for cold-start evaluation**

Partition the unique part identifiers $P$into $K_{\text{outer}}=5$ mutually exclusive folds using shuffled part-level cross-validation.

For each outer fold $j=1,\ldots,5$:

1. Define $P_{\text{train}}^{\left( j \right)}$as the set of training parts.
2. Define $P_{\text{test}}^{\left( j \right)}$as the held-out test parts.
3. Construct the outer training dataset:

$$D_{\text{train}}^{\left( j \right)}=\{(x_{i},y_{i}):\text{part}_{i}\in P_{\text{train}}^{\left( j \right)}\}$$

4. Construct the outer test dataset:

$$D_{\text{test}}^{\left( j \right)}=\{(x_{i},y_{i}):\text{part}_{i}\in P_{\text{test}}^{\left( j \right)}\}$$

All observations belonging to a given part are assigned either to the training set or to the test set, but never both. This ensures that the model is evaluated only on unseen parts.

**Step 3. Cold-start feature construction**

For each outer fold:

1. Generate static metadata features, temporal descriptors, and external covariates for both training and test parts.
2. Generate lagged and rolling demand features only from the training portion of the data.
3. For held-out cold-start test parts, treat all history-dependent features as structurally unavailable.
4. Compute imputation constants for history-dependent features using only $D_{\text{train}}^{\left( j \right)}$.
5. Replace unavailable lagged and rolling features in $D_{\text{test}}^{\left( j \right)}$using the corresponding training-fold imputation constants.
6. Fit all encoders and scalers only on $D_{\text{train}}^{\left( j \right)}$.
7. Apply the fitted encoders and scalers to $D_{\text{test}}^{\left( j \right)}$.

No information from the held-out test parts is used for feature engineering, imputation, encoding, scaling, model training, or hyperparameter tuning.

**Step 4. Inner cross-validation for hyperparameter selection**

Within $D_{\text{train}}^{\left( j \right)}$, partition $P_{\text{train}}^{\left( j \right)}$into $K_{\text{inner}}=3$inner folds.

For each candidate configuration $\theta\in\Theta$:

1. Initialize an empty list of validation errors.
2. For each inner fold $r=1,\ldots,3$:
   - Define inner training parts $P_{\text{inner-train}}^{\left( r \right)}$.
   - Define inner validation parts $P_{\text{inner-val}}^{\left( r \right)}$.
   - Train the candidate model using only observations from $P_{\text{inner-train}}^{\left( r \right)}$.
   - Predict demand for observations from $P_{\text{inner-val}}^{\left( r \right)}$.
   - Compute validation MAE.
   - Store the validation MAE.
3. Compute the mean validation MAE across the three inner folds.

Select the configuration with the lowest mean inner-fold MAE:

$$\theta^{*}=\arg\min_{\theta\in\Theta}\frac{1}{3}\sum_{r=1}^{3} MAE_{r}(\theta)$$

The selected configuration $\theta^{*}$is used only after the inner-loop procedure is complete.

**Step 5. Train final ZIG MC model on the full outer training set**

Using $D_{\text{train}}^{\left( j \right)}$and the selected configuration $\theta^{*}$:

1. Create the binary occurrence target:

$$z_{i}=\left\{ \begin{matrix} 1, & y_{i}>0 \\ 0, & y_{i}=0 \end{matrix} \right.$$

2. Train the occurrence model to estimate the probability of non-zero demand:

$$\hat{p}_{i}=\Pr(y_{i}>0\mid x_{i})$$

3. Train the magnitude model using only observations with positive demand:
4. Estimate the conditional positive-demand mean:

$$\hat{\mu}_{i}=E(y_{i}\mid y_{i}>0,x_{i})$$

5. Parameterize the Gamma distribution using fixed shape $k=2.0$and scale:

$$\hat{\beta}_{i}=\frac{\hat{\mu}_{i}}{k}$$

**Step 6. Generate Monte Carlo predictive samples**

For each observation $i$in $D_{\text{test}}^{\left( j \right)}$:

1. Obtain the predicted probability of demand occurrence $\hat{p}_{i}$.
2. Obtain the predicted positive-demand mean $\hat{\mu}_{i}$.
3. Compute the Gamma scale parameter $\hat{\beta}_{i}=\hat{\mu}_{i}/k$.
4. For each simulation $m=1,\ldots,N_{\text{MC}}$:
   - Draw an occurrence indicator:

$$b_{i}^{\left( m \right)}\sim Bernoulli(\hat{p}_{i})$$

- If $b_{i}^{\left( m \right)}=0$, set:

$$\tilde{y}_{i}^{\left( m \right)}=0$$

- If $b_{i}^{\left( m \right)}=1$, draw:

$$\tilde{y}_{i}^{\left( m \right)}\sim Gamma(k,\hat{\beta}_{i})$$

1. Store the predictive sample set:

$$\tilde{Y}_{i}=\{\tilde{y}_{i}^{\left( 1 \right)},\tilde{y}_{i}^{\left( 2 \right)},\ldots,\tilde{y}_{i}^{\left( N_{\text{MC}} \right)}\}$$

**Step 7. Aggregate predictions and compute metrics**

For each test observation $i$, compute the point forecast as the Monte Carlo mean:

$$\hat{y}_{i}=\frac{1}{N_{\text{MC}}}\sum_{m=1}^{N_{\text{MC}}} \tilde{y}_{i}^{\left( m \right)}$$

Compute probabilistic summaries from $\tilde{Y}_{i}$, including prediction intervals and service-level quantiles.

For each outer fold, compute:

- MAE
- RMSE
- WMAPE
- MASE
- CRPS
- quantile reliability
- fill rate
- stock-out rate
- average backlog
- average inventory level

Store all observed values, point forecasts, predictive samples, and fold-wise metrics.

**Step 8. Final aggregation across outer folds**

After all five outer folds are complete:

1. Concatenate predictions and observed values from all outer folds.
2. Aggregate predictive samples across all held-out test observations.
3. Report the mean and standard deviation of each metric across the five outer folds.
4. Use the aggregated predictive distributions for final probabilistic calibration and inventory analysis.

**Reproducibility safeguards**

The procedure incorporates the following safeguards:

1. All splits are performed at the part level, not at the transaction or observation level.
2. Held-out test parts are never used during training, tuning, imputation, scaling, or encoding.
3. Inner-loop validation is restricted to outer-training parts only.
4. Imputation constants are computed only from the corresponding training fold.
5. A fixed random seed of 42 is used for fold generation, model initialization, and Monte Carlo simulation.
6. Monte Carlo forecasts are generated using $N_{\text{MC}}=100000$simulations for each test observation.
7. Final performance is reported only on parts that are unseen during model fitting and hyperparameter selection.

**S3 Table – Final Optimized Hyperparameters**

Include rows for:

- KNN
- BTM-KNN
- SVR
- Ridge
- Lasso
- CatBoost (single-stage)
- Zero-Inflated two-part model
- Tweedie GLM
- DeepAR
- ZIG MC (classifier + regressor)

| **Model** | **Hyperparameters** |
| --- | --- |
| **KNN** | n_neighbors: 7 (KNN_NEIGHBORS global config), weights: 'distance' |
| **BTM-KNN** | k: 7 (KNN_NEIGHBORS global config), decay_lambda: $\frac{\log(2)}{\text{TEMPORAL\_DECAY\_HALFLIFE\_MONTHS}}$ , embedding_dimension: 6 . |
| **SVR** | kernel: 'rbf' |
| **Ridge** | alpha: 1.0 |
| **Lasso** | alpha: 1.0 |
| **CatBoost** | Regressor: verbose: 0, random_state: 42 (RANDOM_STATE global config)  Classifier: verbose: 0, random_state: 42 (RANDOM_STATE global config) |
| **Zero-Inflated** | Classifier (for zero demand): CatBoostclassifier, random_state: 42. Regressor (for positive demand): CatBoostRegressor, learning_rate: 0.05, n_estimators: 400, random_state: 42. |
| **Tweedie** | power: 1.5, link: 'log', alpha: 0.5, max_iter: 1000 |
| **DeepAR** | learning_rate: 0.001, hidden_size: 128, rnn_layers: 2, dropout: 0.2, loss: NegativeBinomialDistributionLoss() |
| **ZIG MC** | GAMMA_SHAPE: 2.0 (global config), N_SIMULATIONS: 100000 (global config). Inherits parameters from Zero-Inflated two-part model for p_zero and positive_mean. |
