# Supplementary figures and images for: A two stage statistical framework for cold start spare part demand forecasting
Source: PLoS One. 2026 Jun 16;21(6):e0350729. doi: 10.1371/journal.pone.0350729 (PMC13271490; doi:10.1371/journal.pone.0350729)

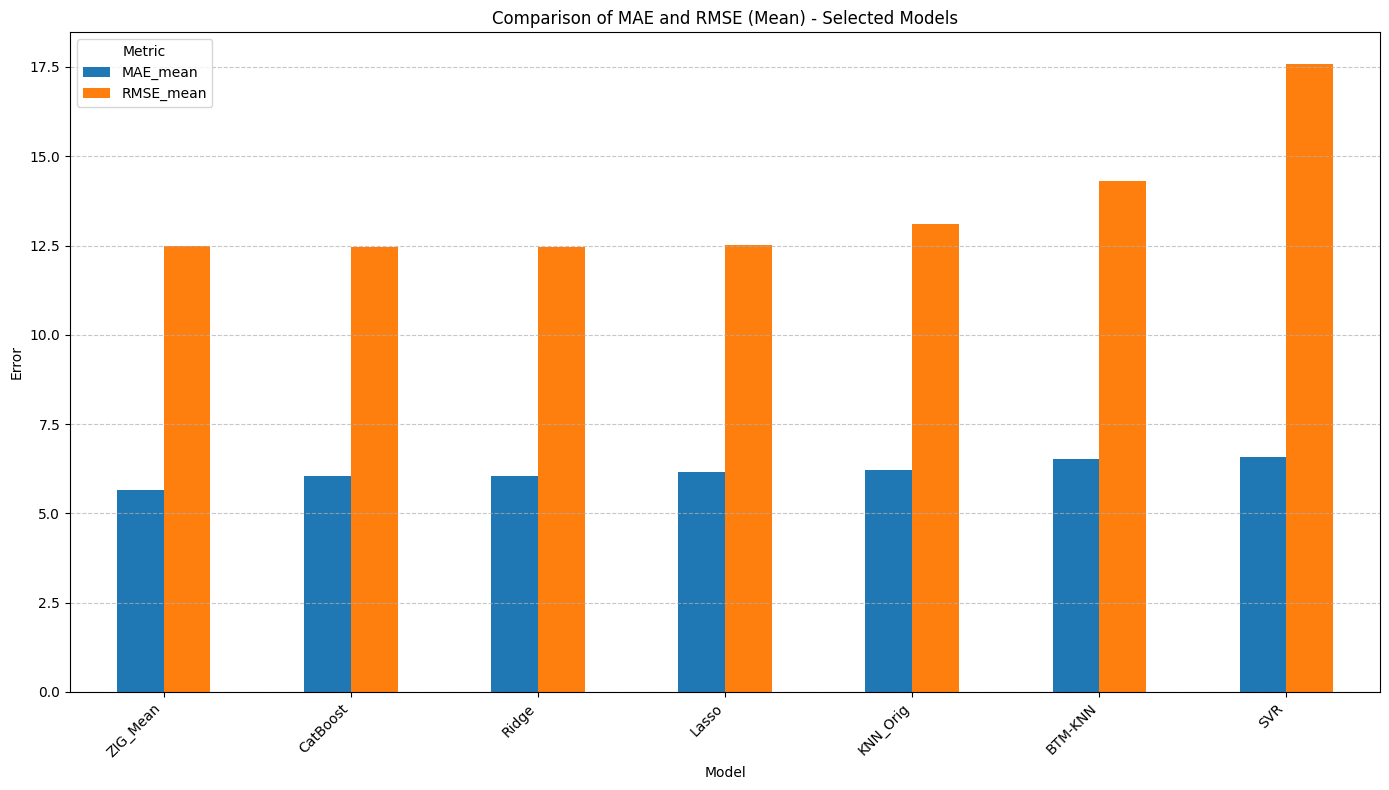

Supplement: S1 Dataset — This file contains the supporting dataset used for model development, validation, and reproducibility of the proposed cold-start spare-part demand forecasting framework. (ZIP) [file pone.0350729.s002.zip › download (8).png]

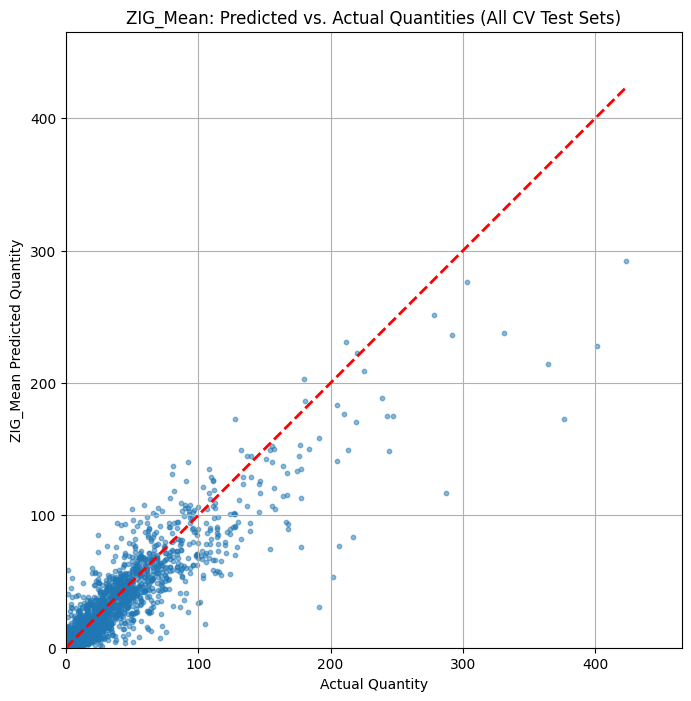

Supplement: S1 Dataset — This file contains the supporting dataset used for model development, validation, and reproducibility of the proposed cold-start spare-part demand forecasting framework. (ZIP) [file pone.0350729.s002.zip › download (11).png]

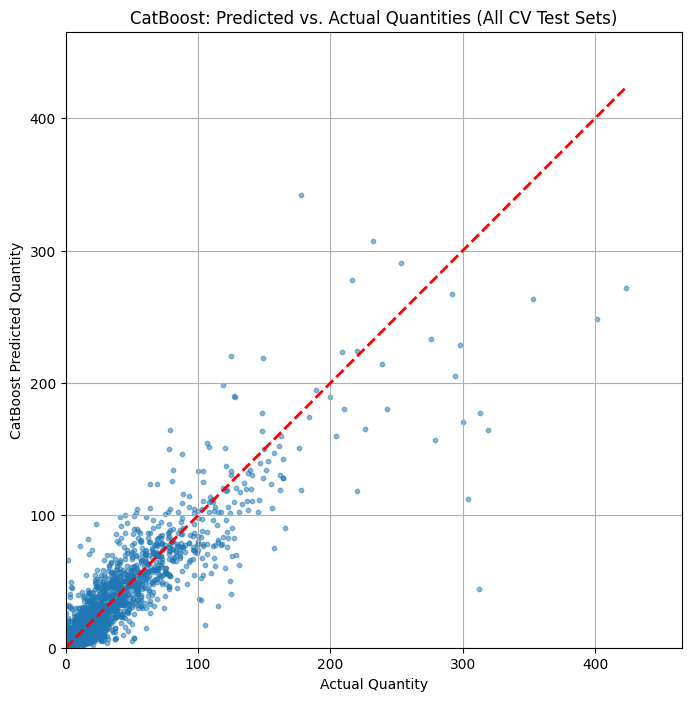

Supplement: S1 Dataset — This file contains the supporting dataset used for model development, validation, and reproducibility of the proposed cold-start spare-part demand forecasting framework. (ZIP) [file pone.0350729.s002.zip › download (14).png]

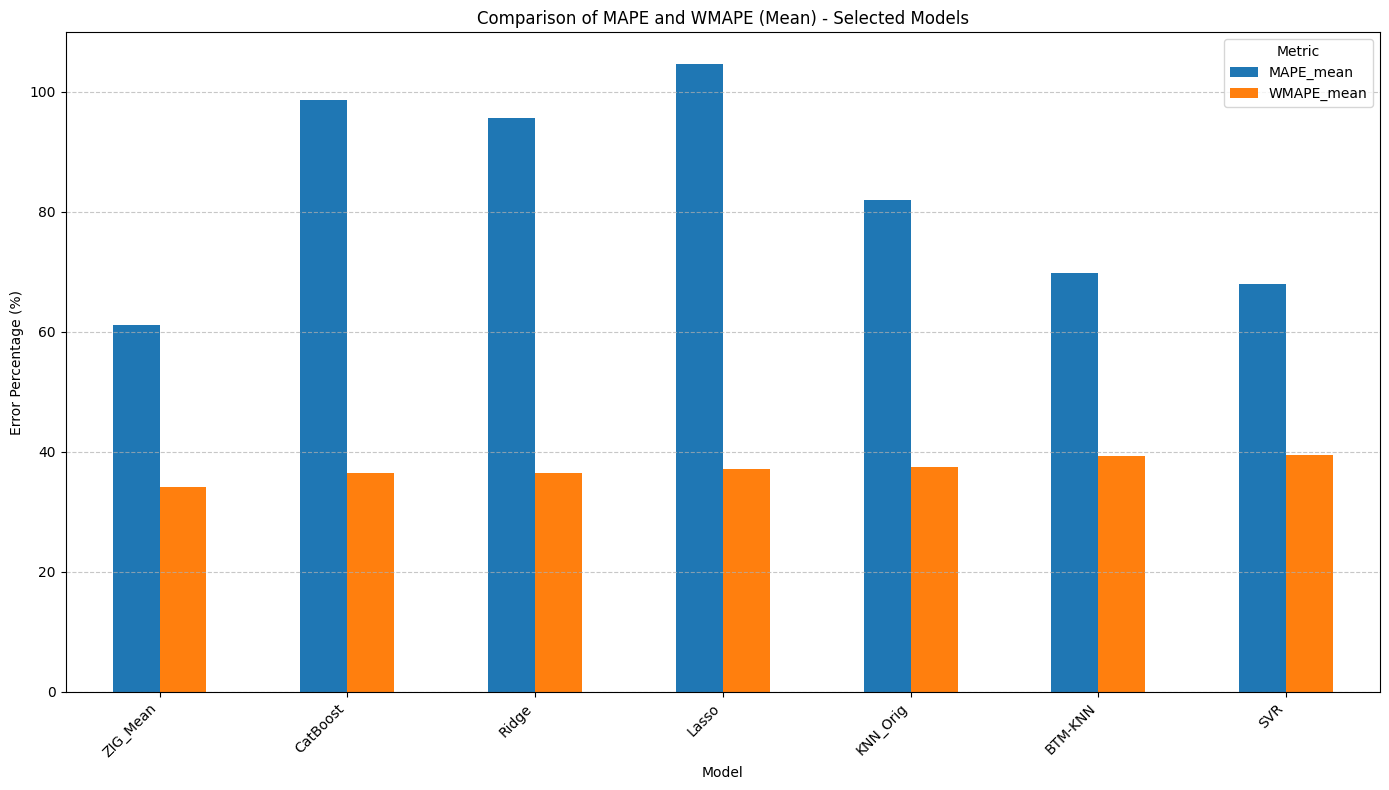

Supplement: S1 Dataset — This file contains the supporting dataset used for model development, validation, and reproducibility of the proposed cold-start spare-part demand forecasting framework. (ZIP) [file pone.0350729.s002.zip › download (9).png]

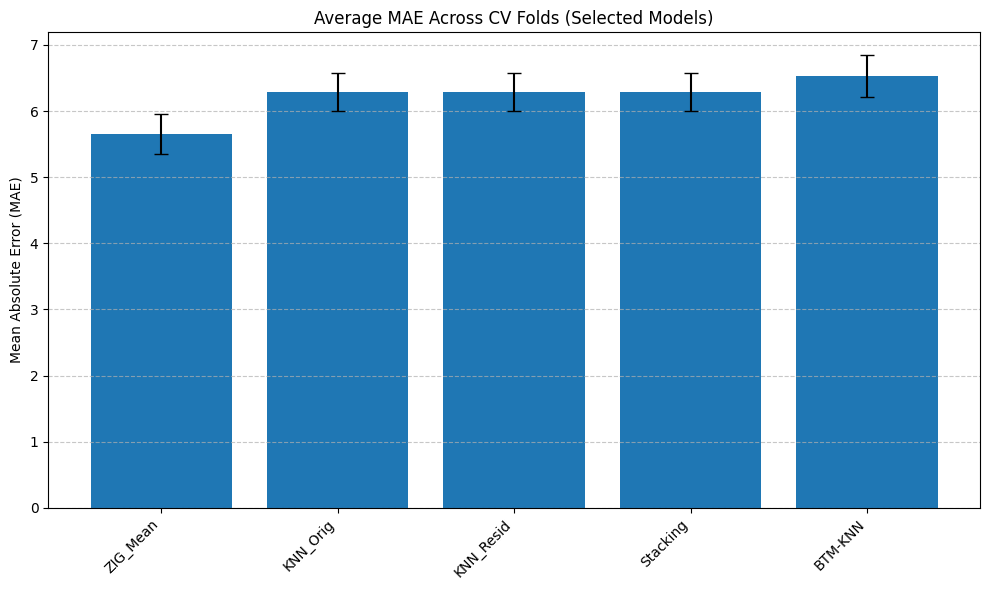

Supplement: S1 Dataset — This file contains the supporting dataset used for model development, validation, and reproducibility of the proposed cold-start spare-part demand forecasting framework. (ZIP) [file pone.0350729.s002.zip › download.png]

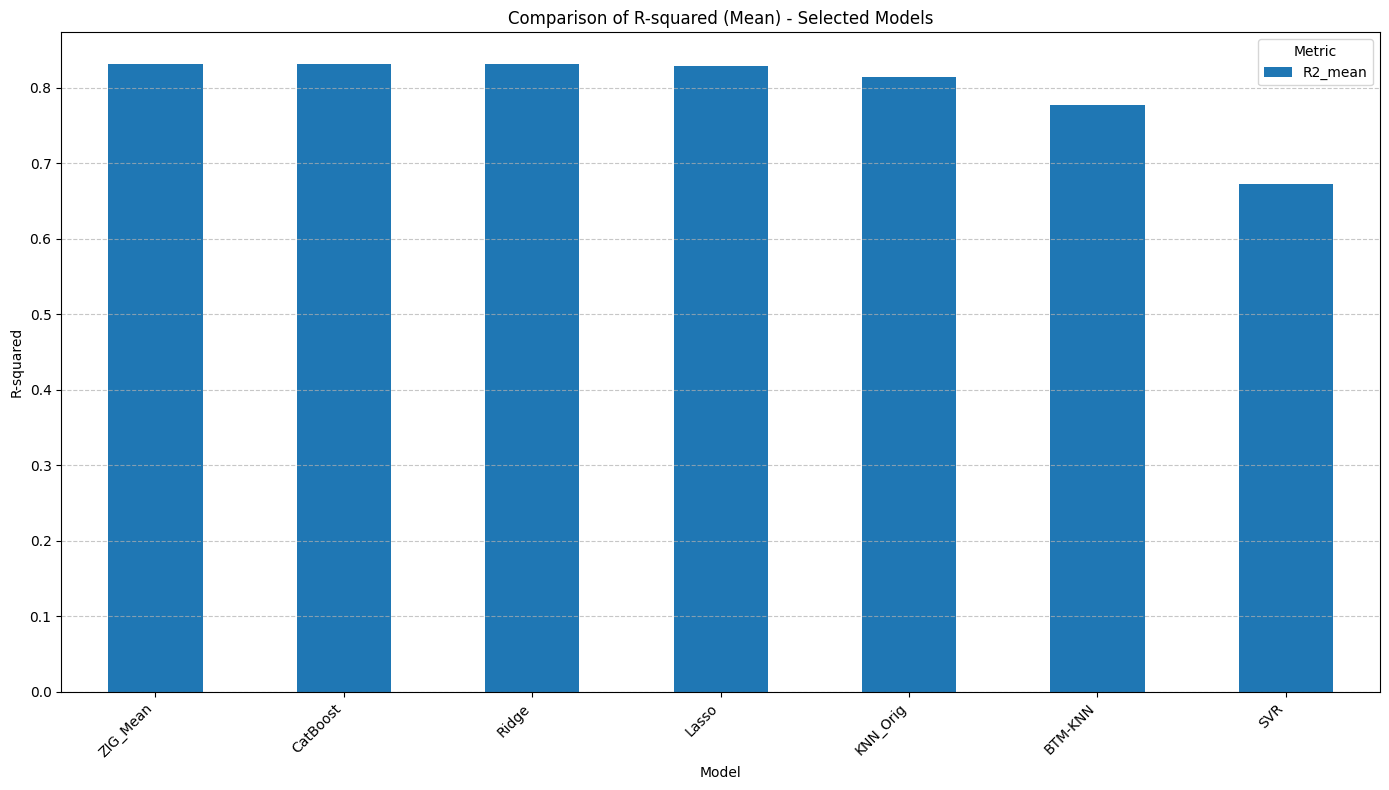

Supplement: S1 Dataset — This file contains the supporting dataset used for model development, validation, and reproducibility of the proposed cold-start spare-part demand forecasting framework. (ZIP) [file pone.0350729.s002.zip › download (10).png]

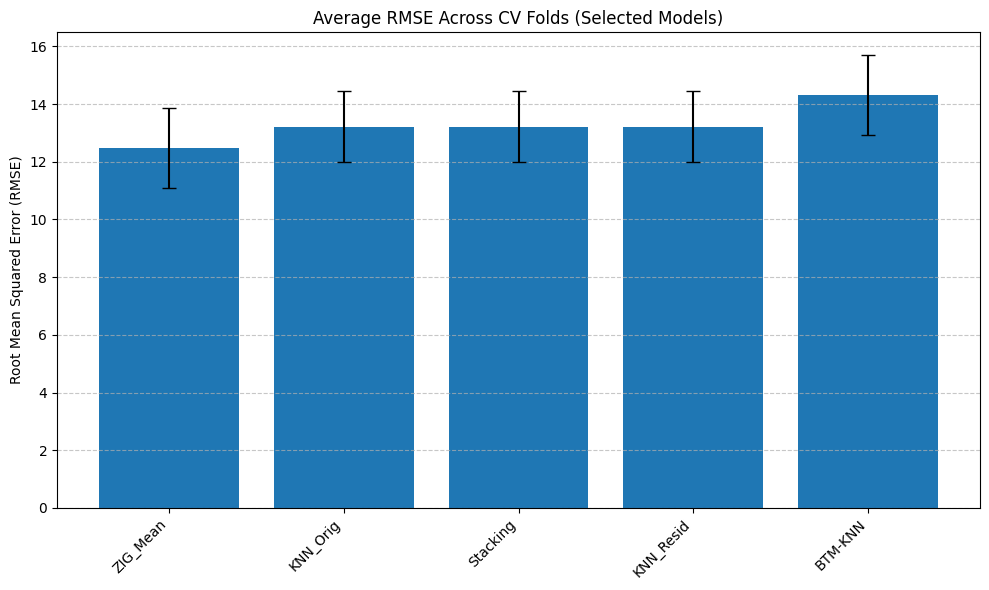

Supplement: S1 Dataset — This file contains the supporting dataset used for model development, validation, and reproducibility of the proposed cold-start spare-part demand forecasting framework. (ZIP) [file pone.0350729.s002.zip › download_1_.png]

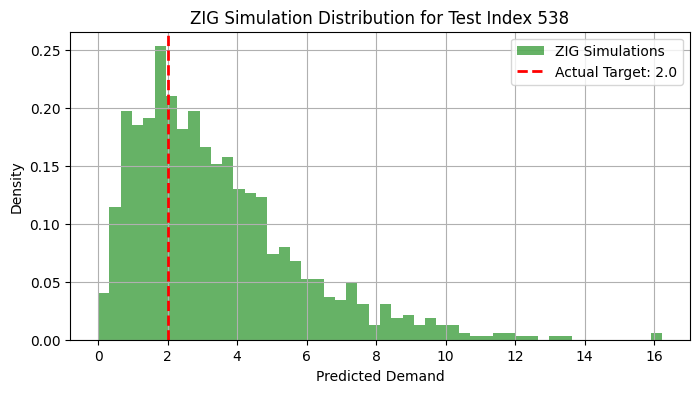

Supplement: S1 Dataset — This file contains the supporting dataset used for model development, validation, and reproducibility of the proposed cold-start spare-part demand forecasting framework. (ZIP) [file pone.0350729.s002.zip › fig_hist1.png]

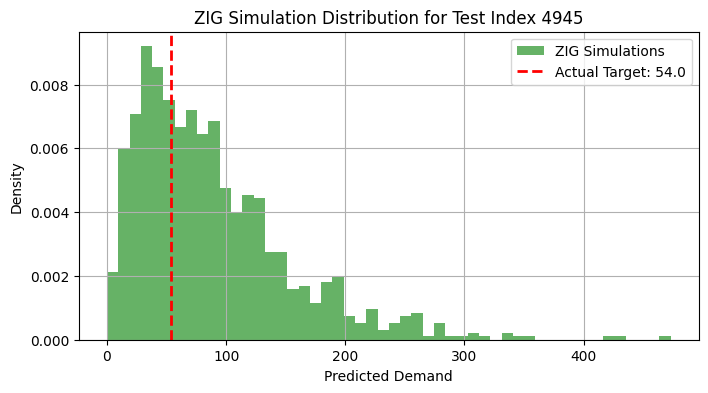

Supplement: S1 Dataset — This file contains the supporting dataset used for model development, validation, and reproducibility of the proposed cold-start spare-part demand forecasting framework. (ZIP) [file pone.0350729.s002.zip › fig_hist2.png]

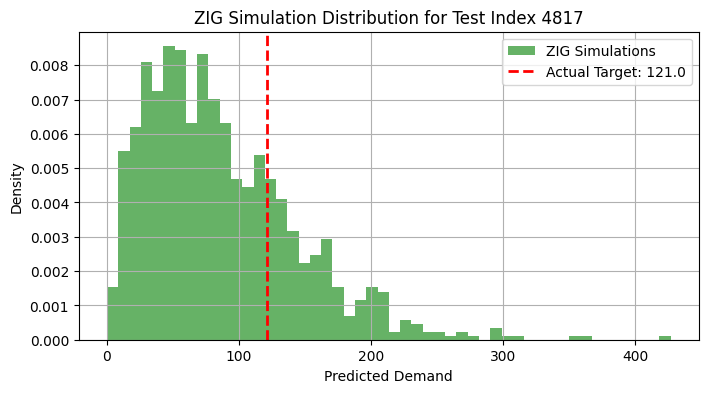

Supplement: S1 Dataset — This file contains the supporting dataset used for model development, validation, and reproducibility of the proposed cold-start spare-part demand forecasting framework. (ZIP) [file pone.0350729.s002.zip › fig_hist3.png]

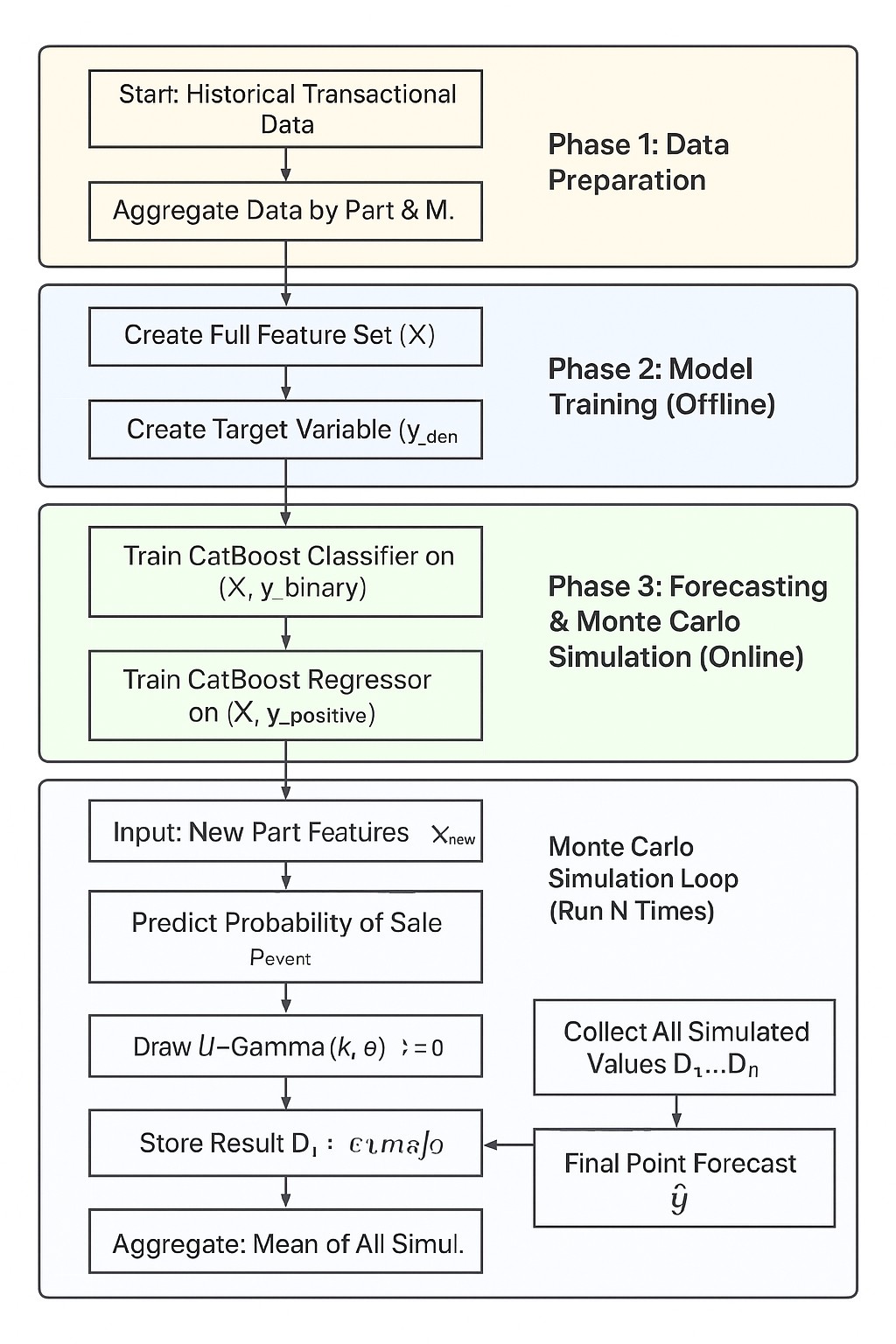

Supplement: S1 Dataset — This file contains the supporting dataset used for model development, validation, and reproducibility of the proposed cold-start spare-part demand forecasting framework. (ZIP) [file pone.0350729.s002.zip › flowchart1.jpg]

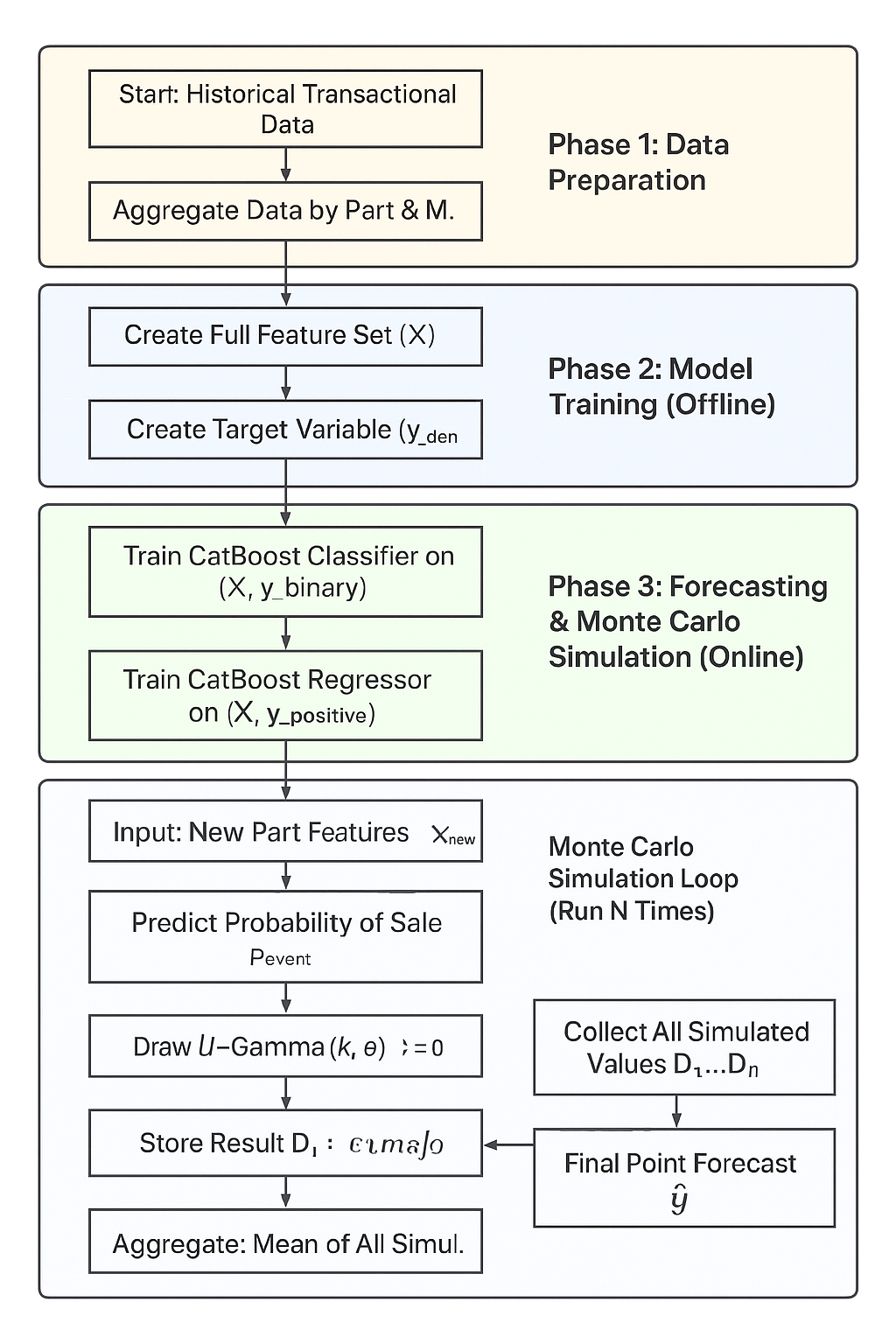

Supplement: S1 Dataset — This file contains the supporting dataset used for model development, validation, and reproducibility of the proposed cold-start spare-part demand forecasting framework. (ZIP) [file pone.0350729.s002.zip › flowchart1.png]
